# TBK1 regulates regeneration of pancreatic β-cells

**DOI:** 10.1038/s41598-020-76600-6

**Published:** 2020-11-09

**Authors:** Yun-Fang Jia, Subbiah Jeeva, Jin Xu, Carrie Jo Heppelmann, Jin Sung Jang, Michael Q. Slama, Subhasish Tapadar, Adegboyega K. Oyelere, Sang-Moo Kang, Aleksey V. Matveyenko, Quinn P. Peterson, Chong Hyun Shin

**Affiliations:** 1 Department of Molecular Pharmacology and Experimental Therapeutics, Mayo Clinic, Rochester, MN 55905 USA; 2 Institute for Biomedical Sciences, Georgia State University, Atlanta, GA 30303 USA; 3 Division of Biology and Biological Engineering, California Institute of Technology, Pasadena, CA 91125 USA; 4 Proteomics Research Center, Mayo Clinic, Rochester, MN 55905 USA; 5 Department of Lab Medicine and Pathology, Mayo Clinic, Rochester, MN 55905 USA; 6 Department of Physiology and Biomedical Engineering, Mayo Clinic, Rochester, MN 55905 USA; 7 School of Chemistry and Biochemistry and the Parker H. Petit Institute for Bioengineering and Bioscience, Georgia Institute of Technology, Atlanta, GA 30332 USA; 8 Center for Regenerative Medicine, Mayo Clinic, Rochester, MN 55905 USA

**Keywords:** Cell biology, Chemical biology

## Abstract

Small-molecule inhibitors of non-canonical IκB kinases TANK-binding kinase 1 (TBK1) and IκB kinase ε (IKKε) have shown to stimulate β-cell regeneration in multiple species. Here we demonstrate that TBK1 is predominantly expressed in β-cells in mammalian islets. Proteomic and transcriptome analyses revealed that genetic silencing of TBK1 increased expression of proteins and genes essential for cell proliferation in INS-1 832/13 rat β-cells. Conversely, TBK1 overexpression decreased sensitivity of β-cells to the elevation of cyclic AMP (cAMP) levels and reduced proliferation of β-cells in a manner dependent on the activity of cAMP-hydrolyzing phosphodiesterase 3 (PDE3). While the mitogenic effect of (*E*)3-(3-phenylbenzo[c]isoxazol-5-yl)acrylic acid (PIAA) is derived from inhibition of TBK1, PIAA augmented glucose-stimulated insulin secretion (GSIS) and expression of β-cell differentiation and proliferation markers in human embryonic stem cell (hESC)-derived β-cells and human islets. TBK1 expression was increased in β-cells upon diabetogenic insults, including in human type 2 diabetic islets. PIAA enhanced expression of cell cycle control molecules and β-cell differentiation markers upon diabetogenic challenges, and accelerated restoration of functional β-cells in streptozotocin (STZ)-induced diabetic mice. Altogether, these data suggest the critical function of TBK1 as a β-cell autonomous replication barrier and present PIAA as a valid therapeutic strategy augmenting functional β-cells.

## Introduction

Replication of pre-existing β-cells was shown to be a major mode of β-cell replenishment maintaining homeostasis during postnatal life in rodents and humans^1–3^. However, the basal rate of β-cell replication declines dramatically with age^4,5^. In mice, β-cells expand their numbers albeit functionally immature during early postnatal stages; subsequently, β-cells acquire the glucose-stimulated insulin secretion (GSIS) capability and lose their proliferative capacity^6–10^. Human β-cells also display a short replicative phase in the postnatal period, peaking in the first year, followed by a long refractory state persisting throughout life^5,11^. Hence, while pieces of evidence indicate the molecular basis for age-related decline in basal β-cell replication^12–15^, further studies are required to better understand molecular targets and signaling pathways regulating the dynamics of proliferation and function of β-cells.

In progression of diabetes, apoptosis triggered by diabetogenic insults, including glucolipotoxicity, leads to decrease in functional β-cells^16,17^. However, the rate of decrease in functional β-cells is much greater than the observed increase in apoptosis in diabetes, therefore, the critical role of loss of molecular identity of β-cells has been accentuated^18,19^. In rodents and humans with chronic, even moderate, hyperglycemia, β-cells fail to maintain a fully differentiated glucose-responsive state^20–25^. Notably, an inverse relation between functional maturation and proliferation of β-cells has been suggested. Replicating β-cells in juvenile mice exhibited reduced levels of genes involved in β-cell maturation and function, especially GSIS^26^. In the *Ins-c-Myc* mouse model expressing c-Myc under the insulin promoter, a pool of β-cells entering the cell cycle displayed decreased expression of β-cell differentiation markers and dysregulated insulin secretion^9^. Unsurprisingly, there are overlaps between immature and dedifferentiated phenotypes at the transcriptome level^21,24,27^. Thus, given that impairment of β-cell function is an early feature of diabetes pathogenesis^28^, delineating the molecular mechanisms and strategies governing the dynamics of proliferation and functionality of β-cells is essential to restore functionally relevant β-cells.

Non-canonical IκB kinases (IKKs), TANK-binding kinase 1 (TBK1) and its homolog IκB kinase ε (IKKε), are key regulators of innate immunity and cancer^29–32^. Moreover, expression of TBK1 and IKKε is induced upon a high-fat diet (HFD) in metabolic tissues to control glucose and energy homeostasis^33–35^. Pharmacological inhibition of TBK1/IKKε promoted energy expenditure in adipose tissue with attenuated hepatic steatosis and improved insulin sensitivity in mouse models of obesity, and enhanced glucose control in a subset of patients with type 2 diabetes (T2D) and non-alcoholic fatty liver disease (NAFLD)^33,36^. Intriguingly, TBK1 and IKKε distinctively control glucose and energy metabolism in response to a HFD. TBK1 inhibits activity of 5′-adenosine monophosphate-activated protein kinase (AMPK), a master sensor of cellular energy status^37–39^, to repress adaptive mitochondrial biogenic response and reduce catabolism^35^. It also controls tumor necrosis factor (TNF)-α-induced nuclear factor (NF)-κB activation in a negative feedback loop^35^. IKKε induces catecholamine resistance in part via activating cyclic AMP (cAMP)-hydrolyzing phosphodiesterase 3B (PDE3B)^40^ to inhibit thermogenic response^33,41^. IKKε has no effect on AMPK phosphorylation and positively regulates inflammation in adipocytes^35^. Thus, adipose-specific deletion of TBK1 (ATKO) attenuates diet-induced obesity with exacerbation in glucose intolerance and insulin resistance, whereas genetic deletion of IKKε increases energy expenditure with improvement in insulin sensitivity on a HFD^34,35^. These data indicate that TBK1 and IKKε use discrete signaling networks to exert their critical effects on regulating glucose and energy metabolism, while showing high sequence homology with comparable phosphorylation profiling of substrate(s)^42^.

Recently, inhibitors of TBK1/IKKε were shown to function as enhancers of β-cell regeneration through a whole organism small molecule screening in a transgenic zebrafish model of type 1 diabetes (T1D) where β-cells are specifically ablated using a chemical-genetic ablation method^43–45^. The most potent β-cell regeneration enhancer was a cinnamic acid derivative (*E*)-3-(3-phenylbenzo[c]isoxazol-5-yl)acrylic acid (abbreviated as PIAA)^45^. PIAA improved glycemic control in streptozotocin (STZ)-induced diabetic mice with increases in insulin content in the pancreas and proliferation of β-cells; it also augmented GSIS and proliferation of β-cells in non-diabetic human islets^45^. However, despite an important function of TBK1/IKKε inhibition in replenishing β-cells^45^, the key question of whether TBK1 and/or IKKε acts as a β-cell autonomous molecular target of PIAA remains elusive. Furthermore, molecular dissection of TBK1- and/or IKKε-controlled signaling networks involved in β-cell proliferation and function as well as regeneration has yet to be investigated.

In this study, we showed that TBK1, not its homolog IKKε, is specifically expressed in β-cells in murine and human islets. Changes in expression of genes and proteins upon genetic silencing of TBK1 in INS-1 832/13 rat β-cells showed increases in the biosynthetic machinery, cell cycle components, and replication markers, indicating that cells were primed for proliferation. Conversely, TBK1 overexpression blunted sensitivity of β-cells to the elevation of cAMP levels and reduced proliferation of β-cells in a manner dependent on the activity of PDE3. While the mitogenic effect of PIAA is derived from inhibition of TBK1, PIAA displayed both pro-proliferation and pro-differentiation potential in human embryonic stem cell (hESC)-derived β-cells and human islets with elevation in insulin secretion. TBK1 expression was induced in β-cells in response to diabetogenic insults, including in human T2D islets. Treatment with PIAA enhanced expression of cell cycle control molecules and β-cell differentiation markers upon diabetogenic challenges, and accelerated restoration of functional β-cells in STZ-induced diabetic mice.

## Results

### TBK1 is specifically expressed in mammalian β-cells and genetic silencing of TBK1 increases β-cell proliferation

**Figure 1:**
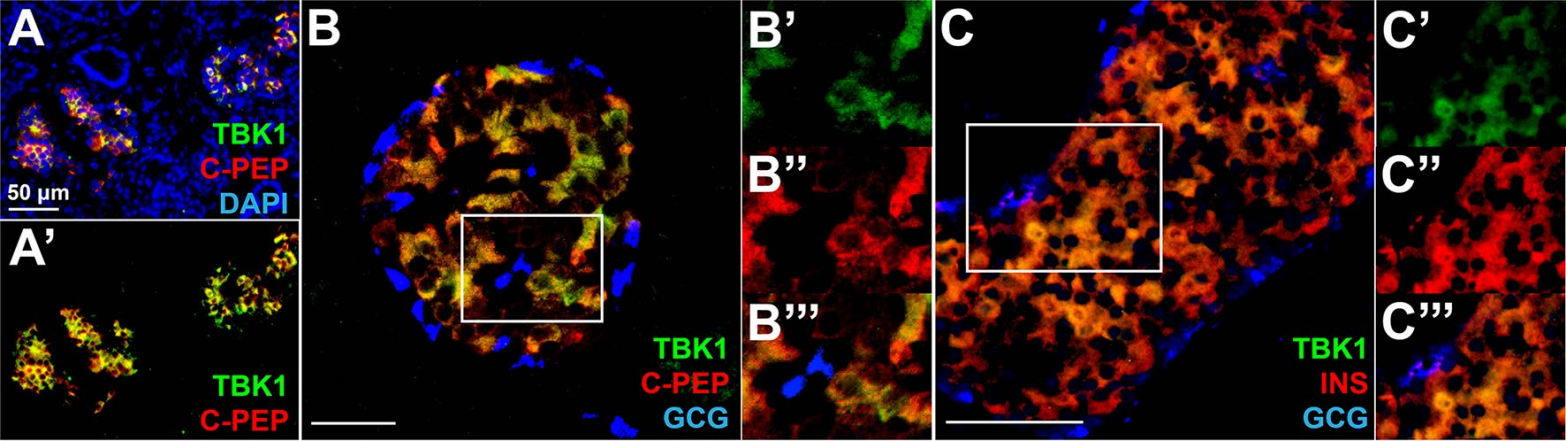
TBK1 is specifically expressed in β-cells in adult human and mouse pancreatic islets. (**A**–**A**′) Confocal images of adult human pancreatic tissues, stained for TBK1 (green), C-Peptide (red), and DAPI (blue). TBK1 is specifically expressed in β-cells in pancreatic islets. (**B**–**B**′′′) Confocal images of adult human pancreatic islets, stained for TBK1 (green), C-Peptide (red), and Glucagon (blue), showing TBK1 expression in β-cells. Magnified images of a white square in (**B**) are shown in (**B**′–**B**′′′). N = 3 donors. (**C**) Confocal images of adult mouse pancreatic islets, stained for TBK1 (green), Insulin (red), and Glucagon (blue). TBK1 is specifically expressed in pancreatic β-cells. (**C**′–C′′′) Magnified images of TBK1 expression in β-cells (a white square in **C**). N = 3 mice. Scale bars: 50 µm.

**Figure 2:**
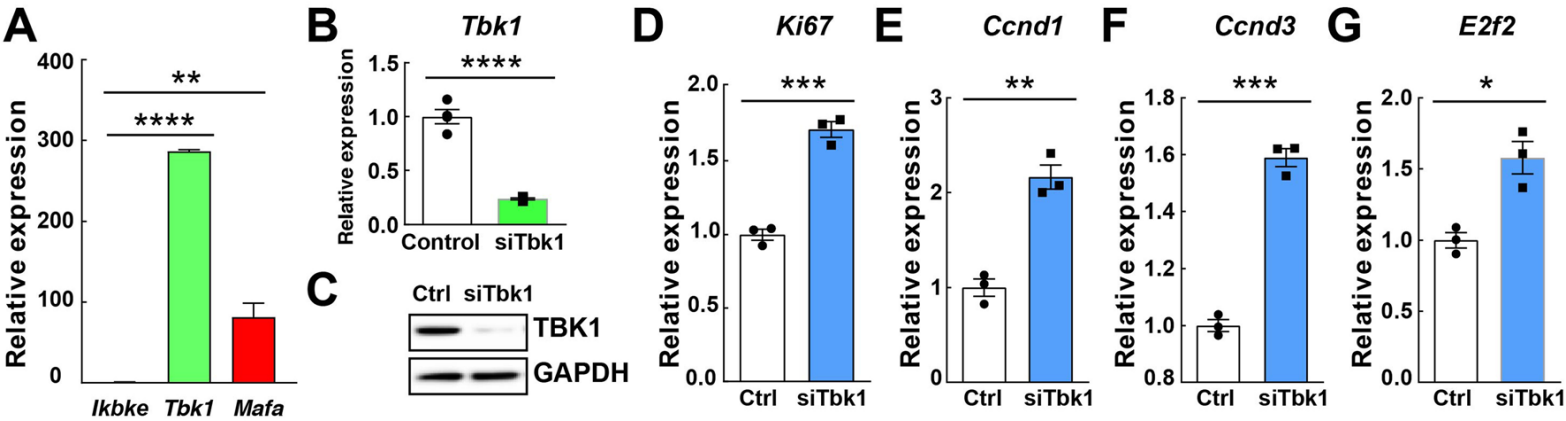
Genetic silencing of *Tbk1* in INS-1 832/13 rat β-cells increases β-cell proliferation. (**A**) Quantitative reverse transcription PCR (RT-qPCR) analysis of *Tbk1*, *Ikbke*, and *Mafa* in INS-1 832/13 β-cells. Quadruplicate. (**B**) RT-qPCR analysis of *Tbk1* in siScramble (control)- and siTbk1-treated INS-1 832/13 β-cells. (**C**) Representative Western blot showing decreased TBK1 proteins levels in siTbk1-treated compared to control INS-1 832/13 β-cells. (**D**–**G**) RT-qPCR analysis of proliferation gene *Ki67* (**D**) and cell cycle regulators *Ccnd1* (**E**), *Ccnd3* (**F**), and *E2f2* (**G**) in control and siTbk1-treated INS-1 832/13 β-cells. Gene expression was normalized to that of *Gapdh* and presented as fold changes (± SEM) against control expression. 3 sample sets per treatment, quadruplicate (**B**) or triplicate (**D**–**G**) per each sample set. Unpaired two-tailed t-test. Asterisk indicates statistical significance: *P < 0.05; **P < 0.01; ***P < 0.001; ****P < 0.0001.

Based on our previous study showing augmented regeneration of β-cells upon treatment with PIAA, a novel pharmacological inhibitor of TBK1/IKKε^45^, we hypothesized that TBK1 and/or IKKε acts as the β-cell autonomous molecular target of PIAA. To test this hypothesis, we performed immunofluorescence analysis to examine the expression of TBK1 and IKKε TBK1 is specifically expressed in β-cells in adult human and mouse pancreatic islets (Fig. 1A–C′′′ and S1), whereas IKKε is distinctively expressed in non-β-cells, primarily in α-cells (Figure S2A–B′′′), in adult human pancreas. Consistent with its confined expression in human and mouse β-cells, *Tbk1* is highly expressed in INS-1 832/13 rat β-cell line (Fig. 2A), a rodent β-cell line that expresses the glucose sensing and insulin-secretory machinery with insulin-secretion function^46^, whereas *Ikbke* expression is nearly undetectable (Fig. 2A).

To assess the role of TBK1 in β-cells, we genetically silenced *Tbk1* using a TBK1-targeting siRNA (siTbk1) in INS-1 832/13 β-cells. A substantial reduction of *Tbk1* mRNA and TBK1 protein upon transfection with siTbk1 (Fig. 2B,C and Fig. S3) led to enhanced expression of proliferation gene *Ki67*, G1/S cell cycle control molecules, including *Ccnd1* (Cyclin D1), *Ccnd3* (Cyclin D3), and *E2f2* (E2F transcription factor 2) (Fig. 2D–G). Together, these data suggest that TBK1 is required for suppressing β-cell replication cell autonomously.

### Proteomic analysis of Tbk1-depleted INS-1 832/13 β-cells displays changes in processes important for cell growth and proliferation

**Figure 3:**
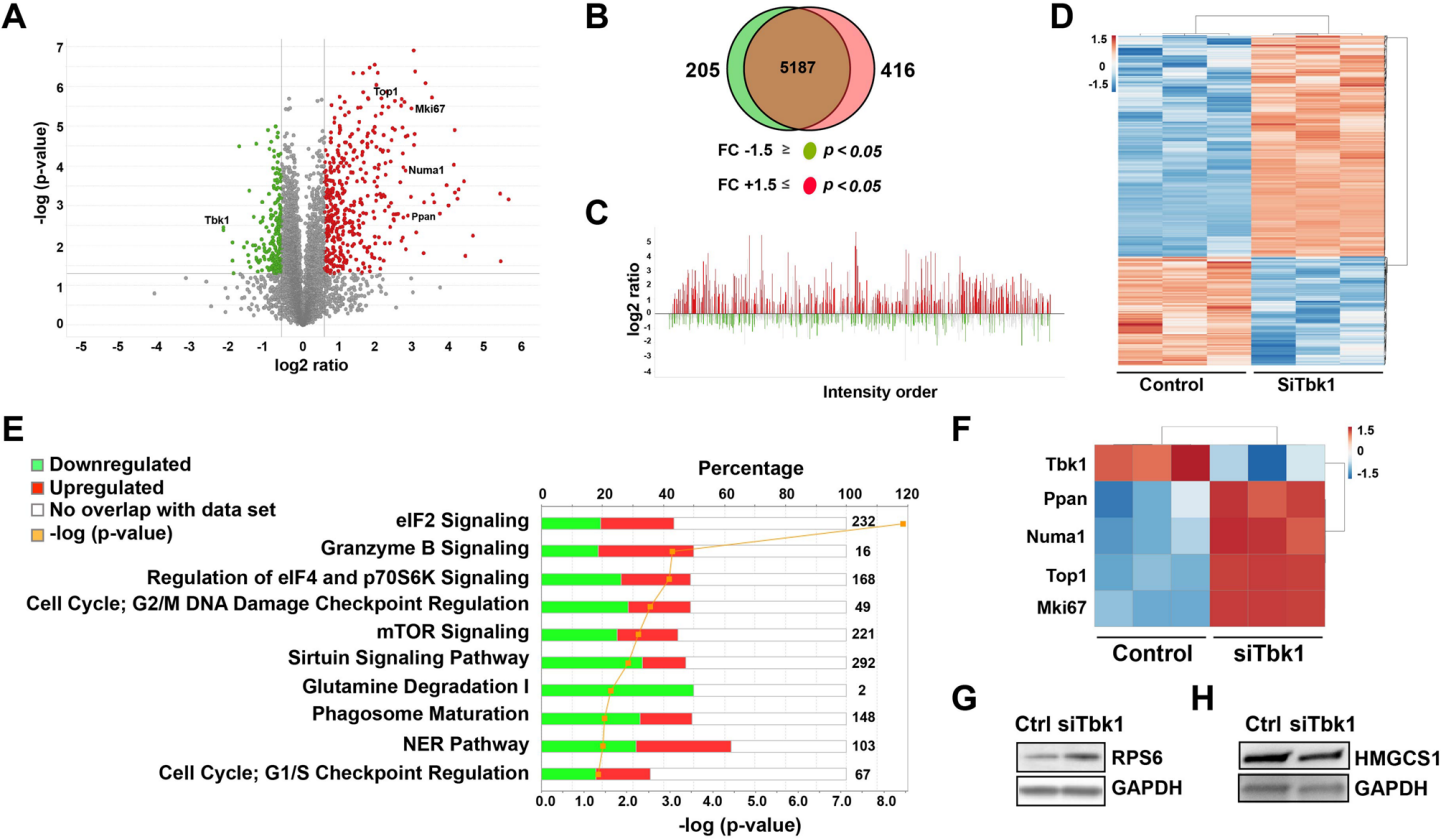
Ingenuity Pathway Analysis identifies top canonical pathways associated with significantly altered focus proteins in *Tbk1*-depleted INS-1 832/13 β-cells. (**A**) Volcano plot displaying the differential expression of proteins cross referenced with *P* value (y-axis; − log_10_) and log_2_ ratio (x-axis). Red and green colors identify at least ± 1.5 fold change and *P* < 0.05, which were defined as significantly altered focus proteins. (**B**) Venn diagram displaying downregulated (green) and upregulated (red) focus proteins in siTbk1-treated relative to siScramble-treated (control) cells. The overlapping brown area in the center of the Venn diagram indicates the number of identified proteins that did not meet focus protein criteria. (**C**) Proteins identified via mass spectrometry using MaxQuant, ordered from most abundant to least abundant. (**D**) Heat map of all focus proteins. (**E**) Ingenuity Pathway Analysis. Orange line indicates the *p*-value of association between reference and focus proteins for each pathway. Numbers on right of pathways indicate the total number of proteins associated with each designated pathway. Red and green indicates % of focus proteins upregulated and downregulated, respectively, in each matched pathway. (**F**) Heat map of several focus proteins important for cell cycle progression in control and *Tbk1*-depleted INS-1 832/13 β-cells. (**G**,**H**) Representative Western blot showing increased RPS6 (**G**) and decreased HMGCS1 (**H**) expression in siTbk1-treated compared to control INS-1 832/13 β-cells.

To uncover mechanisms underlying replicative capacity upon depletion of TBK1, proteomic profiling was conducted on siScramble- and siTbk1-treated INS-1 832/13 β-cells. We identified a total of 5808 proteins and of these, 416 were significantly upregulated and 205 were downregulated upon genetic silencing of TBK1 (Fig. 3A–D).

**Figure 4:**
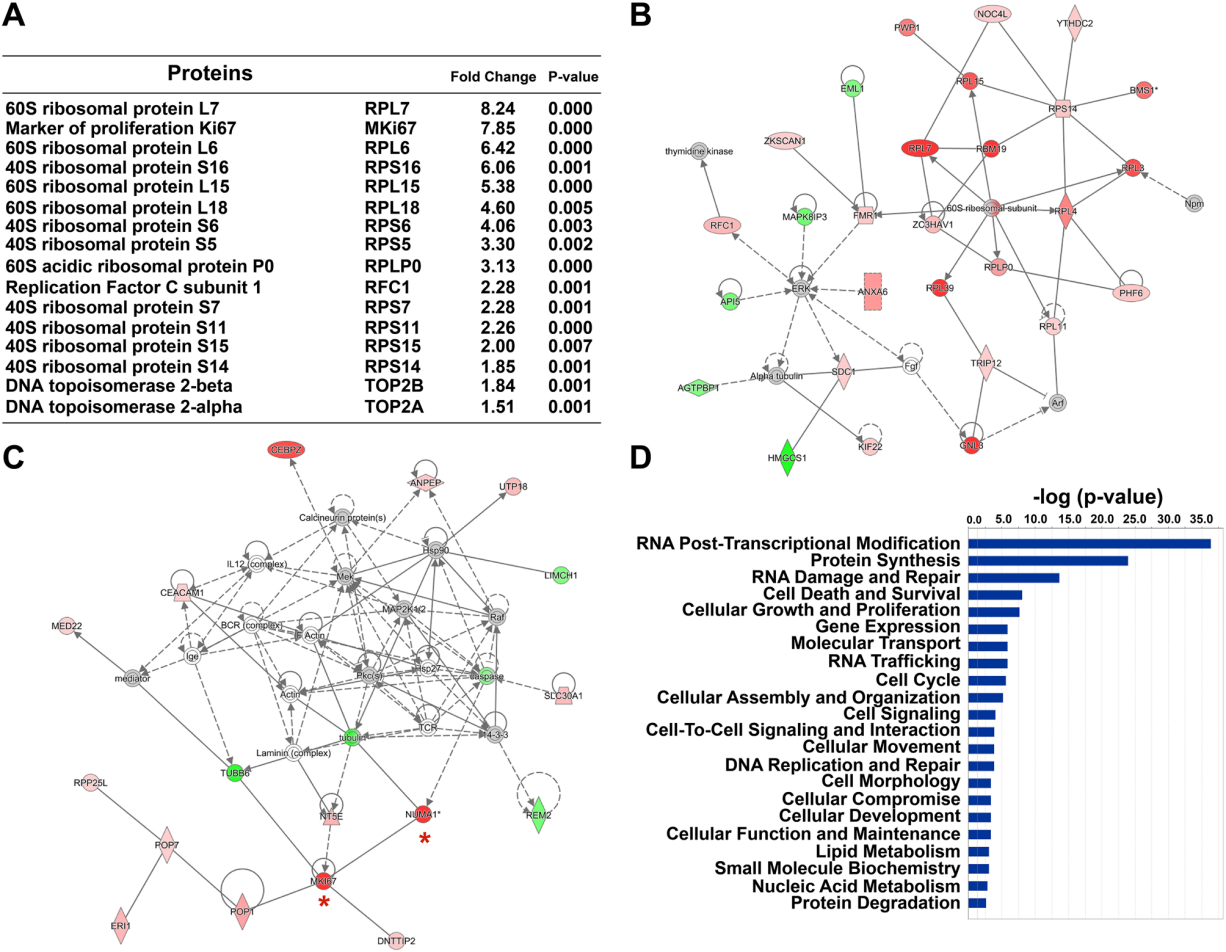
Proteomic analysis of *Tbk1*-depleted INS-1 832/13 β-cells displays changes in processes important for cell growth and proliferation. (**A**) Top focus proteins increased in *Tbk1*-depleted INS-1 832/13 β-cells. (**B**,**C**) Two of the top protein network interactomes generated from focus and reference proteins identified in siTbk1-treated relative to control β-cells, converging onto the assembly of 60S ribosomal subunit (**B**) and MKi67 (**C**; red asterisks). Red and green intensities indicate upregulated and downregulated protein expression, respectively. Gray indicates protein was detected but did not meet focus protein threshold criteria. Solid and dashed line indicate a direct or indirect interaction, respectively. (**D**) Comparison analysis of the top molecular and cellular functions associated with focus proteins in siTbk1-treated β-cells.

Ingenuity Pathway Analysis (IPA) and network interactome analysis revealed that differentially expressed proteins were associated with several signaling pathways, including eukaryotic translation initiation factor 4 (eIF4)/p70S6K (38/168 up and 44/168 down) and mechanistic target of rapamycin (mTOR) (44/221 up and 55/221 down) (Fig. 3E and Supplementary Table 1). Notable increase in the expression of ribosomal proteins for the assembly of 60S and 40S ribosomal subunits is associated with EIF2, eIF4/p70S6K, and mTOR signaling pathways, including an established mTOR complex 1 (mTORC1) target ribosomal protein S6 (RPS6) (Figs. 3E,G, 4A,B, Fig. S4, and Supplementary Table 1). We detected a substantial increase in the expression of proteins involved in G2/M DNA damage checkpoint regulation and nucleotide excision repair (e.g. topoisomerase 1 (TOP1), topoisomerases 2A (TOP2A), topoisomerase 2B (TOP2B), and replication factor C subunit 1 (RFC1)), and cell proliferation (e.g. nuclear mitotic apparatus protein 1 (NUMA1) and marker of proliferation Ki67 (MKi67)) (Fig. 3E,F, 4A,C, and Supplementary Table 1). All of these upregulated proteins are associated with ribosome biogenesis and translation/regulation of translation essential for cell growth, and cell replication. Further analysis of the top physiological processes affected by *Tbk1* deletion in β-cells unveiled a significant number of differentially expressed proteins involved in RNA post-transcriptional modification, protein synthesis, cellular growth and proliferation, cell cycle, and DNA replication, recombination, and repair (Fig. 4D).

Notably, TBK1 depletion led to decrease in expression of proteins associated with transition from immature to adult β-cells, including proteins involved in oxidative phosphorylation, such as mitochondrial complex I (e.g. Ndufa5, Ndufa8, Ndufb5, Ndufb10, Ndufc2, Ndufa12, Ndufb6, Ndufb7, Ndufs8), mitochondrial complex III (e.g. Uqcrq), mitochondrial complex IV (e.g. Cox4i1, Cox5b, Cox6c2), and inner mitochondrial membrane transporters (e.g. Slc25a10, Slc25a13)^7,47^ (Fig. 3E, Fig. S5A–C, Supplementary Table 1, and data not shown). Furthermore, enzymes involved in cholesterol synthesis, including HMG CoA synthase, was downregulated in *Tbk1*-depleted INS-1 832/13 β-cells (Fig. 3H, Fig. S6, and Supplementary Table 1). Consistently, analysis of GSIS demonstrated that siTbk1-treated INS-1 832/13 rat β-cells exhibited a stunted insulin secretory response; they displayed a decline in glucose-stimulated insulin responsiveness (Figure S7A,B). Altogether, these findings indicate that depletion of TBK1 in β-cells upregulates the expression of proteins involved in cell proliferation and biosynthetic processes that are precursors to cell division. Contrarily, genetic silencing of TBK1 downregulates the expression of function-maintaining proteins, which is consistent with the previous studies showing the inverse relation between proliferative capacity and β-cell maturity^9,26^.

### Transcriptome analysis of Tbk1-depleted INS-1 832/13 β-cells reveals a proliferative response

**Figure 5:**
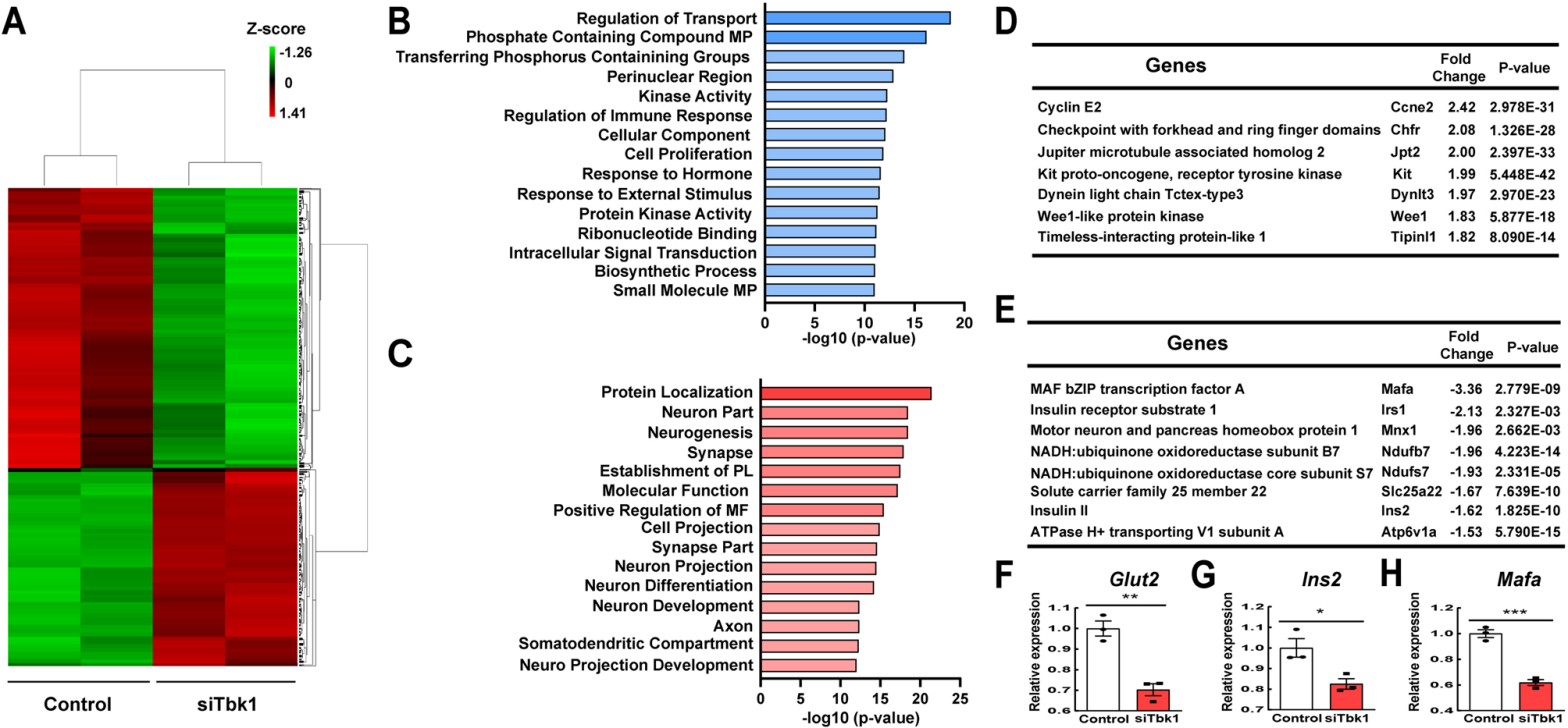
Transcriptome analysis of *Tbk1*-depleted INS-1 832/13 β-cells reveals a proliferative response and diminished maturity. (**A**) Heat map of the genes significantly altered upon *Tbk1* depletion. At least ± 1.5 fold change and a false discovery rate (FDR) cutoff of < 0.05 as well as *P* < 0.05 are defined as significantly altered focus genes. (**B**,**C**) Gene ontology (GO) analysis showing pathways differentially upregulated (**B**) or downregulated (**C**) in siTbk1-treated as compared to control INS-1 832/13 β-cells. (**D**) List of genes showing prominent increase of expression upon *Tbk1* depletion. (**E**) List of genes showing prominent decrease of expression upon *Tbk1* depletion. (**F**–**H**) RT-qPCR analysis of β-cell genes that confer mature features *Glut2* (**F**), *Ins2* (**G**), and *Mafa* (**H**) in control and SiTbk1-treated INS-1 832/13 β-cells. Gene expression was normalized to that of *Gapdh* and presented as fold changes (± SEM) against control expression. 3 sample sets per treatment, triplicate per each sample set. Unpaired two-tailed t-test. Asterisk indicates statistical significance: *P < 0.05; **P < 0.01; ***P < 0.001.

To further unveil mechanisms underlying replicative capacity upon depletion of TBK1, transcriptome profiling was conducted on siScramble- and siTbk1-treated INS-1 832/13 β-cells. A false discovery rate (FDR) cutoff of < 0.05 with a fold change (FC) ± 1.5 revealed downregulation of 587 RNAs and upregulation of 411 RNAs in the siTbk1-treated samples (Fig. 5A). Gene ontology (GO) analysis demonstrated that biosynthetic pathways, including ‘Cell proliferation’ and ‘Biosynthetic process’, among others, were upregulated (Fig. 5B). Accordingly, we observed an increase in expression of multiple genes engaged in proliferation, such as *Ccne2*, *Chfr*, *Jpt2*, *Kit*, *Dyntl3*, *Wee1*, and *Tipinl1* (Fig. 5D). Conversely, genes involved in ‘Protein localization’, ‘Molecular Function’, ‘Positive regulation of molecular function’, among others, were downregulated (Fig. 5C). Thus, downregulation was evident in the expression of β-cell genes that confer mature features, including *Ins2*, *Mafa, Mnx1*, *Irs1*, and genes important for insulin secretion, such as *Ndufs7* and *Ndufb7* (ATP production), *Slc25a22* (mitochondrial glutamate transporter), and *Atp6v1a* (intracellular acidification), in siTbk1-treated cells (Fig. 5E–H). Taken together, these results, along with proteomics analysis, indicate that genetic silencing of TBK1 under basal conditions induces expression of genes involved in proliferation with compromised expression of function-maintaining genes, implicating that TBK1 functions as a key cell autonomous negative regulator of β-cell replication in basal conditions.

### TBK1 regulates proliferation of β-cells via PDE3B

Previous studies suggest that the mitogenic effect of PIAA is mediated at least in part by cAMP-cAMP-dependent protein kinase A (PKA)-mTORC1 signaling cascade via PDE3 in a zebrafish model of T1D and INS-1 832/13 β-cells^45^. Considering these data and mTOR being an evolutionarily conserved serine/threonine protein kinase essential for cell proliferation and survival^48^, and the essential role of TBK1 in suppressing β-cell replication cell autonomously shown above, we hypothesized that TBK1 represses the cAMP-PKA-mTORC1 signaling axis via activation of PDE3. To test this hypothesis, we first analyzed the activities of PKA and mTORC1 upon TBK1 depletion. Phosphorylation of PKA substrate (phospho-Ser/Thr residue of RRXS/T motif) and RPS6 was augmented in siTbk1-treated INS-1 832/13 β-cells (Figures S8A,B, S9, and S10).

Given the previous study showing that PKA directly phosphorylates mTOR and RAPTOR, which are components of mTORC1, in white adipose browning via βAR-cAMP-PKA-mTORC1 signaling pathway^49^, we further tested the possibility whether components of the mTORC1 might be targets of PKA in β-cells. We showed that purified catalytic subunit of PKA (cPKA) phosphorylated mTORC1 component RAPTOR, which was detected with an antibody that recognizes p-PKA substrate, in INS-1 832/13 β-cells (Figures S8C and S11).

Conversely, we overexpressed TBK1 and examined phosphorylation of PKA substrate upon treatment of forskolin, which activates adenylyl cyclase (AC) and raises intracellular cAMP levels. TBK1 overexpression decreased p-PKA substrate levels in response to forskolin (Figures S8D and S12) and downregulated induction of *Ki67* gene expression upon forskolin treatment (Figure S8E). We further examined whether TBK1 would desensitize β-cells to the elevation of cAMP levels through increased activity of PDE3. In reaction to forskolin, PDE3B overexpressing β-cells displayed reduced expression of proliferation gene *Ki67*, while treating PDE3 selective inhibitor cilostamide restored *Ki67* expression in TBK1 overexpressing β-cells (Figures S8E–F and S13), suggesting a critical role for TBK1-PDE3B axis in repressing proliferation of β-cells.

In line with the previous studies demonstrating that PDE3B being predominantly expressed in human and rodent β-cells among PDEs^50^, PDE3B is specifically expressed in adult human pancreatic β-cells along with TBK1 (Figure S8G–G′′′′′). Expression of the other PDE3 family member PDE3A was barely detectable in human pancreatic β-cells (data not shown). Altogether, these data implicate that the TBK1-PDE3B axis suppresses proliferation of β-cells at least in part via repressing cAMP-PKA-mTORC1 signaling cascade (Figure S8H).

### PIAA augments proliferation and function of β-cells

Considering a critical role of TBK1 in suppressing replication of β-cells in a cell autonomous manner shown above, we tested whether PIAA would derive its mitogenic effect via inhibition of TBK1. siTbk1 treatment led to enhanced expression of proliferation gene *Ki67*, while decreased expression of *Mafa* that confers mature features in INS-1 832/13 rat β-cells (Figure S14A,B). PIAA treatment in siTBK1-treated cells increased expression of *Mafa*, while showed minimal effect on expression of *Ki67* (Figure S14A,B). We further explored whether PIAA displays the mitogenic, pro-differentiation, or both effects on β-cells. Treating INS-1 832/13 β-cells with PIAA led to enhanced expression of G1/S cell cycle control molecules, including *Ccnd1* and *Ccnd3*, and β-cell differentiation marker *Mafa* (Figure S14C–E).

**Figure 6:**
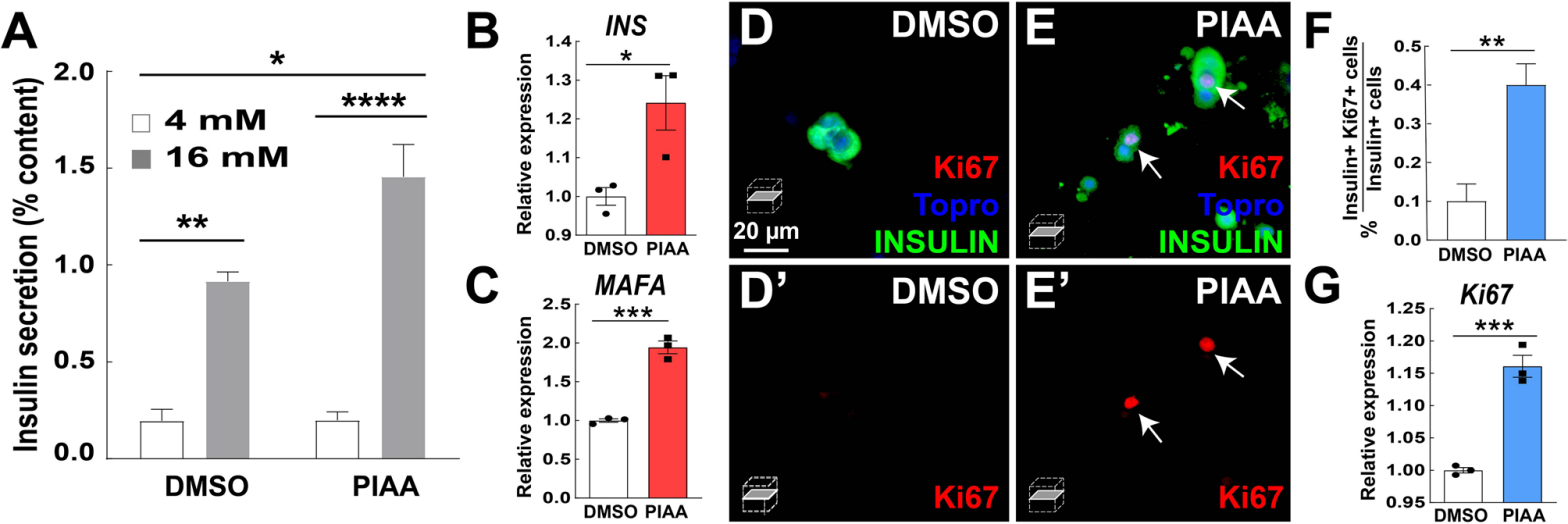
PIAA enhances proliferation and function of β-cells in non-diabetic human islets. (**A**) Glucose stimulated insulin secretion of human islets treated with DMSO or PIAA. Triplicate per condition from 3 cadaveric donors. (**B**,**C**) RT-qPCR analysis of β-cell genes that confer mature features *INS* (B) and *MAFA* (**C**) in DMSO- and PIAA-treated human islets. (**D**–**E**′) Confocal single-plane images of human islets treated with DMSO (**D**,**D**′) and PIAA (**E**,**E**′), respectively, stained for Ki67 (red, white arrows), Topro (blue), and INSULIN (green). Scale bar: 20 μm. (**F**) The percentage (± SEM) of Ki67 and Insulin-double positive cells in human islets in **D**–**E**′. An average of 1391 (vehicle) and 1335 (PIAA) insulin-positive cells were counted (five 96-wells/treatment). (**G**) RT-qPCR analysis of proliferation gene *Ki67* in DMSO- and PIAA-treated human islets. Gene expression was normalized to that of *GAPDH* and presented as fold changes (± SEM) against control expression. 3 sample sets per treatment, triplicate per each sample set. 3 cadaveric donors. Two-way repeated measures ANOVA followed by Bonferroni’s multiple comparisons (**A**) and unpaired two-tailed t-test (**B**,**C** and **F**,**G**). Asterisk indicates statistical significance: *P < 0.05; **P < 0.01; ***P < 0.001; ****P < 0.0001.

To confirm that the dual role of PIAA in enhancing β-cell replication and function is not species specific, we first utilized hESC-derived insulin-producing β-cells to test the effectiveness of PIAA on human β-cells. Consistent with its specific expression in adult human pancreatic β-cells, *TBK1* is highly expressed in hESC-derived β-cells, whereas *IKBKε* expression is barely detectable (Figure S15A). Cells treated with PIAA had enhanced expression of cell cycle control molecule *CCND1* along with β-cell differentiation markers, *INS* and *MAFA*, as compared to vehicle-treated (Figure S15B–D). PIAA treatment in cadaveric human islets increased insulin secretion and led to elevation in expression of β-cell differentiation markers, *INSULIN* and *MAFA* (Fig. 6A–C). It also augmented proliferation of β-cells evaluated by Ki67 staining (Fig. 6D–F) and *Ki67* expression (Fig. 6G). Together, these data suggest that PIAA derives its proliferative effect in large part, if not exclusively, via inhibition of TBK1. Moreover, PIAA can allow both preservation of β-cell differentiated function and increase in β-cell proliferation, not only in rodent β-cells but also in human islets and hESC-derived β-cells, which represent a renewable source of β-cells with a consistent genetic background.

### PIAA enhances replication and function of β-cells in response to diabetogenic insults

**Figure 7:**
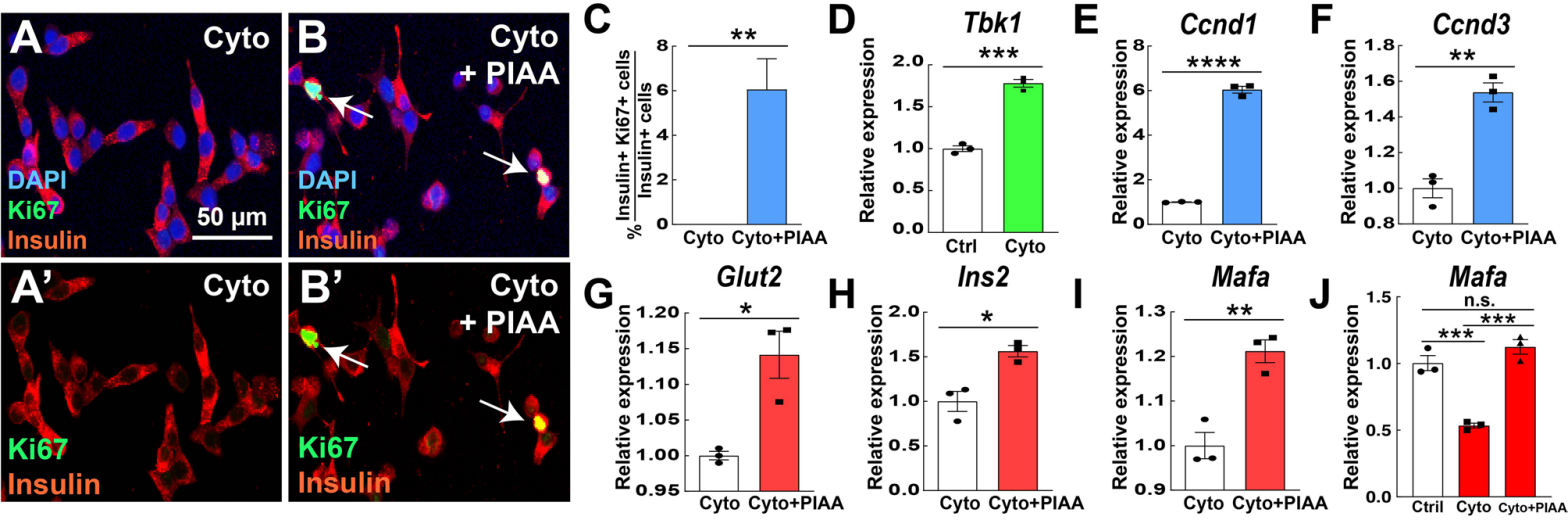
PIAA augments proliferation and expression of cell cycle control molecules and β-cell differentiation markers in response to cytokine-induced diabetogenic challenge. (**A**–**C**) Treatment of PIAA increased the number of Ki67 + Insulin + cells (white arrows; quantified in **C**). A minimum of 560 (cytokines) and 440 (cytokines plus PIAA) insulin-positive cells were counted (5 confocal fields/treatment). Scale bar: 50 μm. (**D**) RT-qPCR analysis of *Tbk1* in control (non-treated) and cytokine-treated INS-1 832/13 β-cells. (**E**–**I**) RT-qPCR analysis of cell cycle regulators *Ccnd1* (**E**) and *Ccnd3* (**F**), and β-cell genes that confer mature features *Glut2* (**G**), *Ins2* (**H**), and *Mafa* (**I**) in cytokine- and cytokine plus PIAA-treated INS-1 832/13 β-cells. (**J**) RT-qPCR analysis of *Mafa* in control (non-treated), cytokine-, and cytokine plus PIAA-treated INS-1 832/13 β-cells. Gene expression was normalized to that of *Gapdh* and presented as fold changes (± SEM) against control expression. 3 sample sets per treatment, triplicate per each sample set. Unpaired two-tailed t-test (**C**–**I**) and one-way ANOVA (**J**). Asterisk indicates statistical significance: *P < 0.05; **P < 0.01; ***P < 0.001; ****P < 0.0001; n.s., not significant.

To further unveil the regenerative potential of PIAA under pathophysiological conditions, we treated INS-1 832/13 cells with a cytokine mix containing IL-1β, IFN-γ, and TNF-α, which are released by inflammatory immune cells and contribute to increased β-cell apoptosis during diabetes progression^51^. While the cytokines increased TBK1 expression in β-cells, PIAA treatment led to enhanced proliferation of β-cells and expression of *Ccnd1* and *Ccnd3* as well as β-cell differentiation markers, including *Glut2*, *Ins2*, and *Mafa*, in cytokine-treated β-cells (Fig. 7A–J).

Multiple injections of low-dose of streptozotocin (MLDS) were shown to result in contributing to β-cell death^52^. TBK1 showed prominently induced expression in MLDS-treated diabetic pancreas (Figure S16A). PIAA administration started causing a substantial reduction of non-fasting blood glucose levels after a week of intraperitoneal injection (Figure S16B). Significant improvement in glucose tolerance was observed compared with vehicle treatment (Figure S16C). Morphometric analysis of pancreas sections showed that the β-cells, not α-cells, in PIAA-treated mice were more likely to be Ki67-positive, indicating that they were proliferating at a higher rate (Figure S16D–F). There was no difference in the weight of the mice based on treatment, neither at the start nor at the end of the experiments (data not shown), indicating that the mice were not generally affected by PIAA treatment.

**Figure 8:**
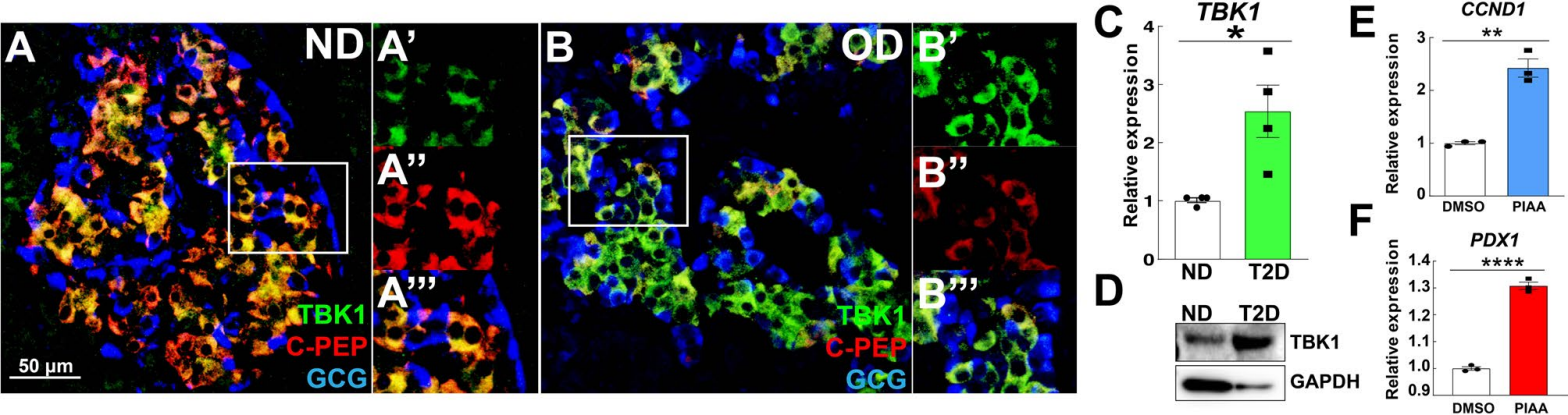
TBK1 expression is elevated in β-cells of patients with type 2 diabetes. (**A**–**B**′′′) Confocal images of adult human pancreatic tissues [**A**–**A**′′′: non-diabetic (ND); **B**–**B**′′′: obese type 2 diabetic (OD)], stained for TBK1 (green), C-Peptide (red), and Glucagon (blue), showing higher TBK1 expression with lower levels of C-Peptide expression in β-cells of type 2 diabetic patients (N = 2 donors) than non-diabetic controls (N = 3 donors). Magnified images of TBK1 expression in β-cells (white squares in **A** and **B**) are shown in **A**′–**A**′′′ and **B**′–**B**′′′, respectively. Scale bar: 50 µm. (**C**) RT-qPCR analysis of *TBK1* mRNA expression in non-diabetic controls (N = 4 donors) versus type 2 diabetic patients (N = 4 donors). Triplicate per donor. (**D**) Representative Western blot showing increased TBK1 expression in type 2 diabetic patient (N = 1 donor). (**E**,**F**) RT-qPCR analysis of cell cycle regulator *CCND1* (**E**) and β-cell gene *PDX1* (**F**) in vehicle- versus PIAA-treated type 2 diabetic islets. Triplicate (N = 1 donor). Gene expression was normalized to that of *GAPDH* and presented as fold changes (± SEM) against control expression; Unpaired two-tailed t test. Asterisks indicate statistical significance: *P < 0.05; **P < 0.01; ****P < 0.0001.

Notably, TBK1 expression was increased in β-cells and pancreatic tissues of T2D patients (Fig. 8A–D and Fig. S17). Treatment of PIAA on human pancreatic islets from T2D patients caused increased expression of cell cycle molecule *CCND1* and β-cell differentiation marker *PDX1* (Fig. 8E,F). Consistently, PIAA treatment under glucolipotoxic condition restored the expression of proliferation genes, including *Ki67* and *Ccnd1*, and β-cell differentiation genes, such as *Mafa* and *Ins2*, which was downregulated by glucolipotoxicity, in INS-1 832/13 rat β-cells (Figure S18A–D). Taken together, these results imply a potential role of TBK1 in repressing a β-cell adaptive response to diabetogenic insults; these data further suggest that PIAA enhances β-cell replication and function across multiple species, including human β-cells, under pathophysiological conditions, thus presenting PIAA as a valid therapeutic strategy augmenting functional β-cells.

## Discussion

In this study, by genetic silencing and proteome as well as transcriptome analyses, we demonstrated a novel role for TBK1 in regulating the dynamics of β-cell proliferation and function. Depletion of TBK1, which is specifically expressed in mammalian β-cells, caused a shift to state poised for proliferation, whereas TBK1 overexpression repressed β-cell replication mediated by the cAMP-PKA-mTORC1 signaling axis via PDE3B. While PIAA derives its mitogenic effect in large part, if not exclusively, via inhibition of TBK1, PIAA exhibited both pro-proliferation and pro-differentiation potential in hESC-derived β-cells and human islets with elevation in insulin secretion. TBK1 expression was induced in β-cells in response to diabetogenic insults, including in human T2D islets. Treatment with PIAA increased expression of cell cycle control molecules and β-cell differentiation markers upon diabetogenic challenges, and promoted restoration of functional β-cells in STZ-induced diabetic mice; these data present PIAA as a valid therapeutic strategy for augmenting functional β-cells.

Our system-wide proteomic and RNA-seq as well as gene expression analyses in INS-1 832/13 rat β-cells suggest that TBK1 plays an important role as a cell autonomous repressor of β-cell replication under basal conditions. In line with its distinct expression in human and mouse β-cells, *Tbk1* is highly expressed in INS-1 832/13 rat β-cells, whereas *Ikbke* expression is nearly undetectable. Genetic silencing of TBK1 in these β-cells led to increased proliferation and reduced expression of β-cell markers consistent with the predicted inverse relation between proliferative capacity and β-cell maturity^9,26^. Thus, it is likely that in physiological conditions, TBK1 acts as one of the proliferation, not functional, barriers of β-cells, which might help to explain the replication-quiescent, not function-quiescent, state of non-diabetic adult β-cells^4,5,53–58^.

Previously, inhibition of activities of PKA and mTOR as well as overexpression of PDE3 blunted the mitogenic effect of PIAA in β-cells in a zebrafish model of T1D and INS-1 832/13 β-cells^45^. Intracellular cAMP levels play pivotal roles in survival and replication of β-cells as well as insulin secretion^59–61^, and are modulated by their rate of synthesis (via adenylyl cyclase) and degradation (via PDEs^62^). Our gain-of-function studies showed that TBK1 dampened sensitivity of β-cells to the elevation of cAMP levels and reduced β-cell proliferation in PDE3 activity-dependent manner. Moreover, proteomics analysis of *Tbk1*-depleted INS-1 832/13 β-cells provided a compelling association between TBK1 silencing and mTORC1 activation. Depletion of TBK1 led to substantial upregulation of ribosomal proteins, which have 5′ terminal oligopyrimidine tract (5′-TOP)^63^. mTORC1 has been suggested to play a fundamental role in controlling mRNA translation, particularly translation of TOP mRNAs bearing a 5′-TOP^63–65^. These TOP mRNAs are the class of transcripts that is most sensitive to translation repression upon mTOR inhibition^66,67^. While mTORC1 has been shown to be important for β-cell function and insulin secretion^68–71^, its critical function in maintaining the proliferative/immature phenotype in adult pancreatic β-cells has been demonstrated^7^. Constitutive activation of mTORC1 from embryonic age to adulthood or inducing mTORC1 in adult β-cells led to increased β-cell proliferation and basal insulin secretion at low glucose concentrations with the concomitant reduction of expression of key β-cell maturation genes in adult β-cells^7^. The immature β-cell phenotype in mTORC1 activation is to a high degree similar to TBK1 depletion phenotype under basal conditions. Furthermore, we demonstrated that PKA directly phosphorylates RAPTOR proteins in β-cells. Mammalian mTOR proteins contain 3 conserved PKA target RRXS motifs and RAPTOR proteins have 1 RRXS motif directly phosphorylated by cPKA in HEK293 cells^49^. Thus, it is plausible that the cell autonomous TBK1-PDE3B axis plays previously unappreciated role in repressing proliferation of β-cells at least in part via suppressing cAMP-PKA-mTORC1 signaling cascade in physiological conditions.

Intriguingly, TBK1 expression increased in β-cells upon diabetogenic insults. Considering the previous studies showing that (1) TBK1 directly phosphorylates AMPK α-subunit to inhibit AMPK activity^35^, (2) TBK1 deficiency upregulated AMPK Thr172 phosphorylation in adipocytes only in response to HFD, not normal diet (ND)^35^, and (3) AMPK is critical for β-cell maturation and function^72–74^, and its activity is reduced in T2D^75,76^, it is worth investigating not only the TBK1-PDE3B axis but also the TBK1-AMPK axis to dissect the TBK1-controlled signaling networks involved in β-cell regeneration, specifically in pathophysiological conditions.

Our data indicate pivotal therapeutic potential of PIAA, which drives proliferation of β-cells via its TBK1 inhibitory activity with additional pro-differentiation effect, for expanding functional β-cells in physiological (rat and hESC-derived β-cells and isolated non-diabetic human islets) and pathophysiological conditions (cytokine- and glucotoxicity-treated β-cells, STZ-induced diabetic mice, and T2D human islets); under pathophysiological conditions, TBK1 expression was induced. Considering our results showing distinctive IKKε expression in non-β-cells (primarily in α-cells) in human islets and low/near undetectable expression in hESC-derived β-cells and rat-derived clonal β-cells, it is crucial to delve into a new paradigm of how non-canonical IκB kinases regulate β-cell proliferation and function as well as regeneration cell autonomously via TBK1 and non-cell autonomously via IKKε. Previously, slightly fewer number of regenerated β-cells were observed in PDE3 inhibitor cilostamide-treated than PIAA-treated T1D zebrafish^45^, which is consistent with our hypothesis that PIAA enhances β-cell regeneration both β-cell autonomously and non-β-cell autonomously. In addition, PIAA treatment during the time window of α-to-β-cell transdifferentiation in T1D zebrafish caused an increase in the number of cells that co-express Insulin and Glucagon, suggesting that PIAA might be able to enhance neogenesis of β-cells from non-β-cells^45^. Hence, elucidation of IKKε-controlled signaling networks involved in regeneration of β-cells and delineation of intracellular signaling pathways mediating the effects of PIAA on β-cell proliferation and function (e.g. by comparing gene/protein profiles of TBK1-depleted vs. PIAA-treated β-cells) need further investigation. Moreover, design/validation of new molecular structures with potent TBK1 and/or IKKε inhibition activities and minimal toxicity using PIAA as a scaffold will allow us to identify legitimate strategies to replenish functionally relevant β-cells in diabetic patients.

## Materials and methods

### INS-1 832/13 rat β-cell culture condition

The rat insulinoma cell line INS-1 832/13 was kindly provided by Dr. C. Newgard (Duke University, Durham, NC) and maintained as previously described^77^. In brief, INS-1 832/13 cells were grown in RPMI 1640 medium supplemented with 10 mM HEPES, 1 mM sodium pyruvate, 100 IU/mL penicillin, 100 mg/mL streptomycin (Invitrogen), 10% heat-inactivated fetal bovine serum (Gemini), and 50 μM β-mercaptoethanol (Sigma) at 37 °C in a humidified 5% CO_2_ atmosphere. For acute stimulation experiments to test the effects of PIAA, INS-1 832/13 cells plated in six-well dishes (~ 70% confluence) were cultured overnight (12–14 h) in serum-free RPMI 1640 medium containing 2.8 mM glucose, 10 mM HEPES, 1 mM pyruvate, 200 U/mL penicillin, 100 μg/mL streptomycin, and 0.1% BSA (fatty acid free, low endotoxin [Sigma; A8806]).

### Human embryonic stem cell culture

HUES8 embryonic stem cells were maintained as previously described^78^. Briefly, cells were adapted to a 3D culture system using spinner flasks and maintained using mTeSR media. Suspension cultures were established by seeding 150 million cells in mTeSR media with 10 μM Y27632 and maintained at 70 rpm in a humidified incubator at 37 °C and 5% CO_2_. Media was changed at 48 h to mTeSR without Y27632. Cells were passaged every 72 h by dispersing to single cells using Accutase and seeded into fresh mTeSR with Y27632. Differentiations were initiated 72 h after seeding into mTeSR. Briefly, cell clusters were allowed to settle to the bottom of the spinner flask and the media was removed by aspiration. Protocol-specific media was introduced to the spinner flask with appropriate growth factors and the flask was returned to the incubator with stirring. PIAA was treated in the final stage of differentiation (at day 25 of protocol) for 96 h.

### Mice experiments

PIAA treatment into mice with multiple injections of low dose streptozotocin (MLDS) was performed as previously described^45^. In brief, eight-week old C57BL/6J male mice (Jackson Laboratory) received daily intraperitoneal injection of 50 mg STZ/kg body weight daily for 5 days. Only mice that had fed blood glucose values of > 300 mg glucose/dL were used. Mice were given daily intraperitoneal injections of vehicle (dimethylsulphoxide final 6.7%; formulated in 0.5% methylcellulose and 0.5% Tween-80) or PIAA (12.5 mg per kg body weight; formulated in 0.5% methylcellulose and 0.5% Tween-80). Mice were housed in pathogen-free facilities and maintained in the Animal Care Facilities at Mayo Clinic, Rochester, MN. Studies conducted and protocols used were approved by the Institutional Animal Care and Use Committee of Mayo Clinic and were in accordance with National Institutes of Health guidelines.

### Glucose-stimulated insulin secretion from human islet culture and INS-1 832/13 rat β-cells

Human islets from donors were purchased from Prodo Laboratories (Irvine, CA) and were cultured in RPMI 1640 medium with 5 mM glucose and 10% fetal bovine serum at 37 °C for 24 h prior to experimentation^45^. Islets were either directly treated with PIAA for 6 days or dissociated with 0.05% trypsin–EDTA (Invitrogen) for 3–5 min with gentle agitation to aid cell cluster disruption. Single cells obtained from islet dissociation were plated and allowed 48 h to attach^79^ prior to treatment with PIAA for 6 days. GSIS was evaluated by static incubation by equilibrating islets with 4 mM glucose in Krebs buffer for one hour. After equilibration, 3 replicates per group were subjected to 1 h sequential incubations with low (4 mM glucose) and high (16 mM glucose). Secreted insulin was monitored using a human ELISA kit (Mercodia) and normalized to total Insulin. GSIS in INS-1 832/13 rat β-cells was performed as previously described^9^.

### Transfection of siRNAs and plasmids, and treatment of cytokines

TBK1 siGENOME SMARTpool siRNA or negative control non-targeting siRNA were transfected into INS-1 832/13 β-cells using DharmaFECT Transfection Reagent (Dharmacon, Lafayette, CO) for 36 h. HA-TBK1 and myc-PDE3B were transfected using FUGENE HD Transfection Reagent (Promega) for 48 h. Cells were seeded at 5 × 10^5^ cells per well in a 6-well plate. Cytokines (IL-1 β, TNF-α, and IFN-γ cocktail) were treated for 48 h.

### Immunohistochemistry

Immunohistochemistry was performed as previously described^45^ using the following antibodies: mouse anti-Glucagon (1:100; Sigma), rabbit anti-TBK1 (1:100; Abcam), rabbit anti-IKKε (1:100; Abcam), rat anti-C-Peptide (1:300; DSHB), rabbit anti-PDE3B (1:100; Cell Signaling), rabbit anti-Ki67 (1:100; Abcam), guinea pig anti-Insulin (1:100; Sigma), and fluorescently conjugated Alexa antibodies (1:200; Molecular Probes). Nuclei were visualized with DAPI (1:2000; Sigma). Islet samples were directly imaged in plates on a Zeiss LSM 700-405 or LSM 780 confocal microscope. Mice pancreata were dissected, fixed in 4% PFA, treated with either 70% ethanol then embedded in paraffin or a 30% sucrose solution then embedded in Tissue-Tek OCT compound (Sakura Finetek). 8 μm-thick sections were obtained by using a cryostat microtome (CryoStar NX70 Cryostat), stained with antibodies, mounted in Vectashield (Vector Laboratories), and imaged on a Zeiss LSM 700-405 confocal microscope.

### Western blot analysis

The rat INS-1 832/13 β-cells were lysed in lysis buffer (50 mM Tris–HCl, pH 7.4, 150 mM NaCl, 1 mM MgCl_2_, 0.5% IGEPAL, freshly added Protease Inhibitor Cocktail and PhosSTOP tablet from Sigma). Proteins were detected with the following antibodies (Cell Signaling): rabbit anti-TBK1 (1:500; product #3013), rabbit anti-Myc (1:500; product #2272), rabbit anti-HA (1:500; product #3724), mouse anti-S6 ribosomal protein (1:1000; product #2317), rabbit anti-phospho-S6 ribosomal protein (Ser240/244) (1:1000; product #5364), rabbit anti-phospho-PKA substrate (RRXS*/T*) (1:1000; product #9624), rabbit anti-HMGCS1 (1:1000, product #42201), and HRP-conjugated secondary antibodies (1:2500). Mouse anti-GAPDH was purchased from Millipore (1:500; MAB 374). I*n vitro* PKA phosphorylation assay was performed as previously described^49^. Briefly, INS-1 832/13 β-cells were transfected with myc-tagged RAPTOR expression plasmid using FUGENE HD (Promega) according to the instructions provided. Cells were collected and lysed, and 2 mg total protein was incubated with anti–c-myc affinity for 4 h (Sigma-Aldrich). Beads were washed twice with the lysis buffer and once with the PKA reaction buffer (40 mM Tris–HCL [pH 7.4], 20 mM magnesium acetate, 0.2 mM ATP), and PKA catalytic subunit was added to the beads. Protein phosphorylation by PKA was measured with rabbit anti-phospho-PKA substrate (RRXS*/T*) antibody described above.

### Glucose tolerance test

For glucose tolerance test, after a 12-h fast, mice were given an intraperitoneal injection of glucose at a dose of 1–2 g per kg body weight. Blood glucose was measured at basal, 30, 60, 90, and 120 min from tail blood using the OneTouch Ultra glucometer (Lifescan).

### Reverse transcription quantitative real-time polymerase chain reaction (RT-qPCR)

RT-qPCR was performed as previously described^80^. In brief, total RNA was extracted using the RNeasy kit (Qiagen). cDNA synthesis was performed using iScript Reverse Transcription Supermix (Bio-Rad). PCR was conducted using iTaq Universal SYBR Green Supermix in triplicate (Bio-Rad). Optimized primers targeting each gene were designed using Primer3 or purchased (Qiagen). The StepONE Plus PCR System (Applied Biosystems) was used to obtain the *C*t value. The relative gene expression of each sample was determined using the comparative *C*t method with *Gapdh* (rat) *GAPDH* (human) as an internal control. The primers used are listed in the Supplementary Table 2.

### Quantification and statistical analysis

All statistical analyses were performed using GraphPad Prism (version 8). Tests used to determine statistical significance were unpaired two-tailed Student’s *t*-test and one-way ANOVA with Tukey’s multiple comparison test (RT-qPCR); two-way ANOVA with Bonferroni’s multiple comparisons (glucose-stimulated insulin secretion assay, glucose tolerance test, and daily glucose measurement). Statistical information for each experiment can be found in the corresponding figure legend. *P* values less than 0.05 were considered statistically significant.

## Supplementary information

Supplementary Information.

## Data Availability

The RNA-seq datasets supporting the current study are being deposited in a public repository (GSE136670).
