# Supplementary material for: TBK1 regulates regeneration of pancreatic β-cells
Source: Sci Rep. 2020 Nov 9;10:19374. doi: 10.1038/s41598-020-76600-6 (PMC7653919; doi:10.1038/s41598-020-76600-6)
Supplement: Supplementary file 1 — Supplementary Information. [file 41598_2020_76600_MOESM1_ESM.pdf]

TBK1 Regulates Regeneration of Pancreatic β-cells

Yun-Fang Jia[1], Subbiah Jeeva[2], Jin Xu[3], Carrie Jo Heppelmann[4], Jin Sung Jang[5], Michael Q. Slama[6], Subhasish Tapadar[7], Adegboyega K. Oyelere[7], Sang-Moo Kang[2], Aleksey V. Matveyenko[6,8], Quinn P. Peterson[6,8], Chong Hyun Shin[1,2,*]

[1] Department of Molecular Pharmacology and Experimental Therapeutics, Mayo Clinic, Rochester, MN 55905, USA

[2] Institute for Biomedical Sciences, Georgia State University, Atlanta, GA 30303, USA

[3] Division of Biology and Biological Engineering, California Institute of Technology, Pasadena, CA 91125, USA

[4] Proteomics Research Center, Mayo Clinic, Rochester, MN 55905, USA

[5] Department of Lab Medicine and Pathology, Mayo Clinic, Rochester, MN 55905, USA

[6] Department of Physiology and Biomedical Engineering, Mayo Clinic, Rochester, MN 55905, USA

[7] School of Chemistry and Biochemistry and the Parker H. Petit Institute for Bioengineering and Bioscience, Georgia Institute of Technology, Atlanta, GA 30332, USA

[8]Center for Regenerative Medicine, Mayo Clinic, Rochester, MN 55905, USA

*Correspondence: cshin@gsu.edu

Supplementary Information

Supplemental Experimental Procedures

Label free proteomics

Gel electrophoresis and trypsin digest: The supernatants of siScramble- and siTbk1-treated lysates were extracted and protein concentration was determined using Bradford assay, and 20 μg of each sample was loaded onto a 4-12% Bis-Tris poly-acrylamide gel (Invitrogen, Carlsbad, CA) for electrophoresis. Gel was stained with BioSafe Coomassie according to directions supplied by company (BioRad, Hercules, CA), each gel lane was divided into 6 segments down the length of the lane. Each gel segment (6 per sample) was excised, cut into 1-2 mm pieces and transferred to 0.5 ml tubes for in-gel digest. Proteins were destained with 40% acetonitrile with 50mM Tris (pH 8.1) until clear, reduced with 50 mM TCEP in 50 mM Tris (pH 8.1) for 40 minutes at 60°C, followed with alkylation using 25 mM iodoacetamide in 50 mM Tris (pH 8.1) for 40 minutes in the dark at room temperature. Proteins were digested in-situ with 0.16 μg trypsin (Promega Corporation, Madison WI) in 25 mM Tris pH 8.1 and 0.0002% Zwittergent 3-16, overnight at 37°C, followed by peptide extraction with 4% trifluoroacetic acid and acetonitrile. Extractions were dried and stored at -20°C.

Mass spectrometry: Dried trypsin digested samples were suspended in 0.2% formic acid/0.1% TFA/0.002% zwittergent 3-16. A portion of the sample was analyzed by nano-flow liquid chromatography electrospray tandem mass spectrometry (nanoLC-ESI-MS/MS) using a Thermo Scientific Q-Exactive Mass Spectrometer (Thermo Fisher Scientific, Bremen, Germany) coupled to a Thermo Ultimate 3000 RSLCnano HPLC

system. The digest peptide mixture was loaded onto a 330 nL Halo 2.7 ES-C18 trap (Optimize Technologies, Oregon City, OR). Chromatography was performed using A solvent (98% water/2% acetonitrile/0.2 % formic acid) and B solvent (80% acetonitrile/10% isopropanol/10% water/0.2% formic acid), over a 2% to 45% B gradient for 90 minutes at 400 nL/min through a PicoFrit (New Objective, Woburn, MA) 100 µm x 33 cm column hand packed with Agilent Poroshell 120 EC C18 packing (Agilent Technologies, Santa Clara, CA). Q-Exactive mass spectrometer was set to acquire an ms1 survey scans from 350-1600 m/z at resolution 70,000 (at 200 m/z) with an AGC target of 3e6 ions and a maximum ion inject time of 60 msec. Survey scans were followed by HCD MS/MS scans on the top 15 ions at resolution 17,500 with an AGC target of 2e5 ions and a maximum ion inject time of 60 msec. Dynamic exclusion placed selected ions on an exclusion list for 40 seconds.

Data analysis: MaxQuant software (Max Planck Institute of Biochemistry, Martinsried, Germany), version 1.6.0.16, was used for database search, time align, and peak extract information from generated mass spectrometry files[1]. Label free parameters within the MaxQuant software were used to generate normalized protein intensities reported in a protein groups table. Perseus software (Max Planck Institute of Biochemistry, Martinsried, Germany), version 1.6.2.1, was used to perform differential expression of identified proteins[2]. Briefly, protein intensities were log2 transformed, missing values were replaced from a normal distribution, Student's *t*-test was performed requiring valid values to be present in all samples of at least one group, and p-values and q-values were reported. Protein groups with a $p < 0.05$ and a fold change of at least 1.5 were considered as significantly differentially expressed.

RNA-seq and data analysis

200 ng of total RNA from each sample was used. The RNA-Seq libraries were generated using TruSeq Stranded mRNA Library Prep kit (Illumina, CA) at Medical Genome Facility according to the manufacturer's protocol. Constructed libraries were quantified by BioAnalizer 2100 system using D1000 kit (Agilent, CA) and Qubit dsDNA BR Assay kits (Thermo Fisher Scientific, MA). All libraries were pooled and sequenced 101 bp paired-end reads on Illumina HiSeq 2500 Rapid Run. FASTQ files were uploaded into Partek Flow software (Partek Inc., MO) and performed primary QC. The STAR (2.6.1d) aligner was used to perform alignment of reads to the rn6 build of the *Rattus norvegicus* full reference genome. After alignment, the final BAM files were quantified using Partek E/M algorithm[3] by Ensembl annotations (rn6 ensemble release96 v2). DESeq2 package[4] was used to normalize data and determine differential expression of RNA-Seq between two groups. The false discovery rate (FDR) by Benjamin and Hochberg method was used to adjust for comparison. FDR value less than 0.05 and fold change ±1.5 at raw *P* value less than 0.05 were considered as significant change. Hierarchical clustering was performed on the significantly changed genes based on the Euclidean distance and the average linkage clustering algorithm using Partek Flow software. To further understand the biological meaning of the data set, Gene Set Enrichment Analysis (GSEA, Broad Institute) was carried out in Molecular Signatures Database v5.2 (MsigDB).

Ki67-postive cell counting

Human islets

To calculate the number of proliferating β-cells in dissociated cultured human islets, the total number of Ki67+ and INSULIN+ double positive cells in each well was manually counted using a Zeiss LSM 700-405 confocal microscope. The total number of INSULIN+ cells in each well was estimated approximately by multiplying the number of INSULIN+ cells in a single cluster of islet and the total number of clusters with INSULIN+ cells.

STZ-induced diabetic mice

To calculate the number of proliferating β-cells, images of >10 islets in two 8 μm-thick pancreas sections (separated by 400 μm) per animal were taken using a Zeiss LSM 700-405 confocal microscope. The number of Ki67+ and Insulin+ double positive cells, and the total number of Insulin+ cells in the same islet were counted with ImageJ (20 planes of confocal images; N = 3 mice per group).

Supplemental References

1 Tyanova, S., Temu, T. & Cox, J. The MaxQuant computational platform for mass spectrometry-based shotgun proteomics. *Nat. Protoc.* **11**, 2301-2319, doi:10.1038/nprot.2016.136 (2016).

2 Tyanova, S. *et al.* The Perseus computational platform for comprehensive analysis of (prote)omics data. *Nat. Methods* **13**, 731-740, doi:10.1038/nmeth.3901 (2016).

3 Xing, Y. *et al.* An expectation-maximization algorithm for probabilistic reconstructions of full-length isoforms from splice graphs. *Nucleic Acids Res.* **34**, 3150-3160, doi:10.1093/nar/gkl396 (2006).

4 Love, M. I., Huber, W. & Anders, S. Moderated estimation of fold change and dispersion for RNA-seq data with DESeq2. *Genome Biol.* **15**, 550, doi:10.1186/s13059-014-0550-8 (2014).

Supplemental Figure Legends

Supplementary figure 1. Quantification of colocalization of TBK1 with C-Peptide and Glucagon. Total 681 (C-Peptide-positive), 587 (TBK1-positive), and 197 (Glucagon-positive) cells were counted.

Supplementary figure 2. IKKε is specifically expressed in non-β-cells, primarily in α-cells, in adult human pancreatic islets. (A-A') Confocal images of adult human pancreatic tissues, stained for IKKε (green), C-Peptide (red), and DAPI (blue). IKKε is expressed in non-β-cells in pancreatic islets. (B-B''') Confocal images of adult human pancreatic islets, stained for IKKε (green), C-Peptide (red), and Glucagon (blue), showing IKKε expression in non-β-cells, primarily in α-cells. Magnified images of a white square in B are shown in B'-B'''. N = 3 donors. Scale bars: 50 μm.

Supplementary figure 3. Full western blot scans for Figure 2C. The full scans for each blot are shown right.

Supplementary figure 4. Full western blot scans for Figure 3G. The full scans for each blot are shown right.

Supplementary figure 5. Proteomic analysis of *Tbk1*-depleted INS-1 832/13 β-cells displays decrease in expression of proteins involved in β-cell maturity. (A) Top focus proteins decreased in *Tbk1*-depleted INS-1 832/13 β-cells. (B-C) Two top protein

network interactomes generated from focus and reference proteins identified in siTbk1-treated relative to control β-cells, converging onto vacuolar H+-ATPase (B; ATP6V1A, ATP6V1B2, ATP6V1E1; green asterisks) and mitochondrial complex 1 (C; NDUFB10, NDUFC2; green asterisks). Red and green intensities indicate upregulated and downregulated protein expression, respectively. Gray indicates protein was detected but did not meet focus protein threshold criteria. Solid and dashed line indicate a direct or indirect interaction, respectively.

Supplementary figure 6. Full western blot scans for Figure 3H. The full scans for each blot are shown right.

Supplementary figure 7. Genetic silencing of TBK1 leads to dysregulated insulin secretion. (A) Glucose-stimulated insulin secretion (GSIS) under basal (2.8 mM glucose) followed by stimulatory (16.7 mM) conditions in siScramble (control)- and siTbk1-treated INS-1 832/13 rat β-cells. (B) Fold change in GSIS in INS-1 832/13 rat β-cells treated with siTbk1 as compared to control. 3 sample sets per treatment, triplicate per each sample set. All values, mean ± SEM. Two-way ANOVA (A) or unpaired two-tailed t-test (B). Asterisk indicates statistical significance: *, $P < 0.05$; **, $P < 0.01$; n.s., not significant.

Supplementary figure 8. TBK1-PDE3B axis regulates proliferation of β-cells via cAMP-PKA and mTORC1 signaling cascade. (A-B) Representative Western blot showing increased phosphorylation of PKA substrate (A) and mTORC1 target RPS6 (B)

in siTbk1-treated INS-1 832/13 β-cells. (C) myc-RAPTOR were transfected into INS-1 832/13 β-cells for 48 hours, followed by immunoprecipitation from cell lysates with anti–c-Myc–conjugated beads and incubation with PKA catalytic (cPKA) subunit. Western blot analysis was performed with anti-p-PKA substrate (phospho-Ser/Thr residue of RRXS/T motif) and anti-Myc antibodies. (D) Representative Western blot analysis of whole cell lysates from INS-1 832/13 β-cells expressing empty vector or HA-TBK1, and treated with or without 25 μM forskolin for 30 min, showing decreased p-PKA substrate levels in TBK1 overexpressing cells. (E) RT-qPCR analysis of proliferation gene *Ki67* in INS-1 832/13 β-cells expressing empty vector, HA-TBK1, or myc-PDE3B, and treated with or without 10 μM cilostamide or 25 μM forskolin for 30 min. One-way ANOVA. Asterisk indicates statistical significance: *, $P < 0.05$; ***, $P < 0.001$; ****, $P < 0.0001$. (F) Representative Western blot analysis of whole cell lysates from INS-1 832/13 β-cells expressing myc-PDE3B. (G-G’’’’’) Confocal images of adult human pancreatic islets, stained for TBK1 (green), PDE3B (blue), and C-Peptide (red). TBK1 and PDE3B are specifically expressed in pancreatic β-cells. Magnified images of TBK1, PDE3B, and C-Peptide expression in β-cells (white squares in G and G’) are shown in G’’-G’’’’’. N = 3 donors. Scale bar: 50 μm. (H) Schematic of the hypothetical TBK1-PDE3 axis that modulates cAMP-PKA-mTORC1 signaling cascade for proliferation of β-cells. The sites of inhibition by PIAA and cilostamide are shown in red; activation by forskolin (Fsk) in black arrow.

Supplementary figure 9. Full western blot scans for Supplementary Figure 8A. The full scans for each blot are shown right.

Supplementary figure 10. Full western blot scans for Supplementary Figure 8B. The full scans for each blot are shown right.

Supplementary figure 11. Full western blot scans for Supplementary Figure 8C. The full scans for each blot are shown right.

Supplementary figure 12. Full western blot scans for Supplementary Figure 8D. The full scans for each blot are shown right.

Supplementary figure 13. Full western blot scans for Supplementary Figure 8F. The full scans for each blot are shown right.

Supplementary figure 14. PIAA derives its mitogenic effects primarily via inhibition of TBK1 and augments expression of cell cycle control molecules and β-cell differentiation markers in INS-1 832/13 β-cells. (A-B) RT-qPCR analysis of proliferation gene *Ki67* (A) and *Mafa* (B) in siScramble (control)-, siTbk1-treated, and siTbk1 plus PIAA-treated INS-1 832/13 β-cells. (C-E) RT-qPCR analysis of cell cycle regulators *Ccnd1* (C) and *Ccnd3* (D), and *Mafa* (E) in DMSO- and PIAA-treated INS-1 832/13 β-cells. Gene expression was normalized to that of *Gapdh* and presented as fold changes (±SEM) against control expression. 3 sample sets per treatment, triplicate per each sample set. One-way ANOVA (A-B) and unpaired two-tailed t-test (C-E). Asterisk indicates statistical significance: *, $P < 0.05$; **, $P < 0.01$; ***, $P < 0.001$; ****, $P <$

0.0001; n.s., not significant.

Supplementary figure 15. PIAA enhances expression of cell cycle control molecules and β-cell differentiation markers in human embryonic stem cell (hESC)-derived β-cells. (A) RT-qPCR analysis of *TBK1* and *IKBKE* in hESC-derived β-cells. (B-D) RT-qPCR analysis of cell cycle regulator *CCND1* (B) and β-cell genes that confer mature features *INS* (C) and *MAFA* (D) in DMSO- and PIAA-treated hESC-derived β-cells. Gene expression was normalized to that of *GAPDH* and presented as fold changes (±SEM) against control expression. 3 sample sets per treatment, triplicate per each sample set. Unpaired two-tailed t-test. Asterisk indicates statistical significance: *, $P < 0.05$; ***, $P < 0.001$; ****, $P < 0.0001$.

Supplementary figure 16. PIAA improves glucose control and β-cell mass in the STZ-induced diabetic mouse model. (A) RT-qPCR analysis of *Tbk1* in STZ-induced mice (N =3 mice per group for pancreas; N = 4 mice per group for liver). (B-F) STZ-induced diabetic mice were treated with vehicle or PIAA for 3 weeks after reaching > 300 mg/dL fed glucose values. (B) PIAA caused reduction of hyperglycemia (non-fasting glucose measurement) relative to vehicle-treated animals. N = 5 mice per group. (C) PIAA-treated animals showed improved glucose tolerance. diabetic: N = 7 mice; diabetic plus PIAA: N = 6 mice. (D-E) Confocal images of diabetic pancreata treated with vehicle (D) and PIAA (E), respectively, stained for Ki67 (red, white arrows), Insulin (green), and Glucagon (blue). (F) The percentage (± SEM) of Ki67 and Insulin-double positive cells in diabetic islets with or without PIAA treatment in D and E. A minimum

of 829 (diabetic) and 959 (diabetic plus PIAA) insulin-positive cells were counted (10 islets/treatment). Two-way repeated measures ANOVA followed by Bonferroni's multiple comparisons (B and C) and Unpaired two-tailed t-test (F). Asterisk indicates statistical significance: ****, $P < 0.0001$.

Supplementary figure 17. Full western blot scans for Figure 8D. The full scans for each blot are shown right.

Supplementary figure 18. PIAA restores the expression of proliferation genes and β-cell differentiation markers under glucolipotoxic condition. RT-qPCR analysis of proliferation gene *Ki67* (A) and cell cycle regulator *Ccnd1* (B), and β-cell maturation genes *Mafa* (C) and *Ins2* (D) in low glucose (5 mM), glucolipotoxic (25 mM glucose plus 0.2 mM palmitate, 48-hour treatment), and glucolipotoxic plus PIAA-treated INS-1 832/13 rat β-cells. 3 sample sets per treatment, triplicate per each sample set. All values, mean ± SEM. One-way ANOVA. Asterisk indicates statistical significance: *, $P < 0.05$; **, $P < 0.01$; ***, $P < 0.001$; ****, $P < 0.0001$; n.s., not significant. LG, low glucose; HGL, high glucose and lipotoxic.

Supplementary Table 1. Top Focus Proteins and Associated Canonical Pathways Identified in *Tbk1*-depleted INS-1 832/13 β-cells

Name	Description	Canonical pathway	Fold change	*P* value
RPL11	60S ribosomal protein L11	EIF2, eIF4/p70S6K, mTOR signaling	1.56	4.519E-04
RPL4	60S ribosomal *protein* L4	EIF2, eIF4/p70S6K, mTOR signaling	3.89	1.056E-05
RPL3	60S ribosomal *protein* L3	EIF2, eIF4/p70S6K, mTOR signaling	5.62	2.356E-03
RPL39	60S ribosomal *protein* L39	EIF2, eIF4/p70S6K, mTOR signaling	8.29	1.569E-05
RPS29	40S ribosomal protein S29	EIF2, eIF4/p70S6K, mTOR signaling	4.79	1.671E-04
RPS11	40S ribosomal protein S11	EIF2, eIF4/p70S6K, mTOR signaling	2.26	2.557E-05
RPL7	60S ribosomal *protein* L7	EIF2, eIF4/p70S6K, mTOR signaling	8.24	2.963E-05
RPL15	60S ribosomal *protein* L15	EIF2, eIF4/p70S6K, mTOR signaling	5.38	2.251E-05
RPS7	40S ribosomal protein S7	EIF2, eIF4/p70S6K, mTOR signaling	2.28	1.191E-03

RPS6	40S ribosomal protein S6	EIF2, eIF4/p70S6K,<br><br>mTOR signaling	4.06	3.292E-04
RPS15	40S ribosomal protein S15	EIF2, eIF4/p70S6K,<br><br>mTOR signaling	2.0	1.596E-03
RPS16	40S ribosomal protein S16	EIF2, eIF4/p70S6K,<br><br>mTOR signaling	6.06	6.626E-04
RPL6	60S ribosomal *protein* L6	EIF2, eIF4/p70S6K,<br><br>mTOR signaling	6.42	3.031E-6
RPS5	40S ribosomal protein S5	EIF2, eIF4/p70S6K,<br><br>mTOR signaling	3.30	1.532E-04
RPL18	60S ribosomal *protein* L18	EIF2, eIF4/p70S6K,<br><br>mTOR signaling	4.60	5.325E-04
RPS14	40S ribosomal protein S14	EIF2, eIF4/p70S6K,<br><br>mTOR signaling	1.85	1.198E-03
RPLP0	60S acidic ribosomal protein P0	EIF2, eIF4/p70S6K,<br><br>mTOR signaling	3.13	1.401E-06
TOP2A	DNA topoisomerase 2-alpha	Cell cycle	1.51	7.986E-05
TOP2B	DNA topoisomerase 2-beta	Cell cycle	1.84	9.794E-04
RFC1	Replication Factor C Subunit 1	NER pathway	2.28	8.427E-05
MKi67	Marker of proliferation Ki-67		7.85	3.567E-06
NDUFB10	NADH:ubiquinone oxidoreductase subunit B10	Sirtuin signaling	-1.74	3.184E-04

ATP6V1A	ATPase H+ transporting V1 subunit A	Phagosome maturation	-1.64	1.855E-05
ATP6V1B2	ATPase H+ transporting V1 subunit B2	Phagosome maturation	-1.53	1.654E-03
ATP6V1E1	ATPase H+ transporting V1 subunit E1	Phagosome maturation	-1.68	1.009E-05
HMGCS1	3-hydroxy-3-methylglutaryl-coA synthase 1	Mevalonate pathway	-3.36	3.192E-05
SLC25A10	*Solute Carrier Family 25 Member 10*		-1.50	1.673E-04

Supplementary Table 2. PCR primers used for RT-qPCR

Name	Sequence
CCND1 forward	TCTACACCGACAACTCCATCCG
CCND1 reverse	TCTGGCATTTTGGAGAGGAAGTG
CCND3 forward	AGATCAAGCCGCACATGCGGAA
CCND3 reverse	ACGCAAGACAGGTAGCGATCCA
Ki67 forward	TCCTTTGGTGGGCACCTAAGACCTG
Ki67 reverse	TGATGGTTGAGGTCGTTCCTTGATG
INS forward	TCACACCTGGTGGAAGCTCTCTA
INS reverse	ACAATGCCACGCTTCTGCAGGGAC
MAFA forward	GAGCGGCTACCAGCATCAC
MAFA reverse	CTCTGGAGTTGGCACTTCTCG
GAPDH forward	GTCTCCTCTGACTTCAACAGCG
GAPDH reverse	ACCACCCTGTTGCTGTAGCCAA
Rat *Ki67* forward	ATTTCAGTTCCGCCAATCC
Rat *Ki67* reverse	GGCTTCCGTCTTCATACCTAAA
Rat *Mafa* forward	AGGAGGTCATCCGACTGAAACA
Rat *Mafa* reverse	GCGTAGCCGCGGTTCTT
Rat *Ins2* forward	GGTTCTCACTTGGTGGAAGCTC
Rat *Ins2* reverse	GTGCCAAGGTCTGAAGGTCAC
Rat *Glut2* forward	TAGTCAGATTGCTGGCCTCAGCTT
Rat *Glut2* reverse	TTGCCCTGACTTCCTCTTCCAACT

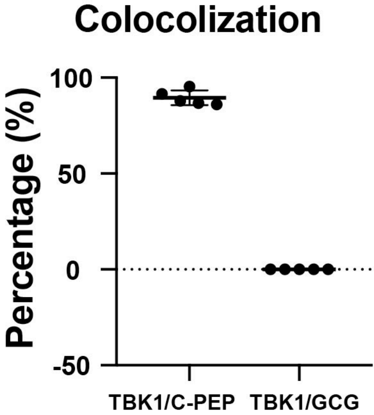

Supplementary Figure 1

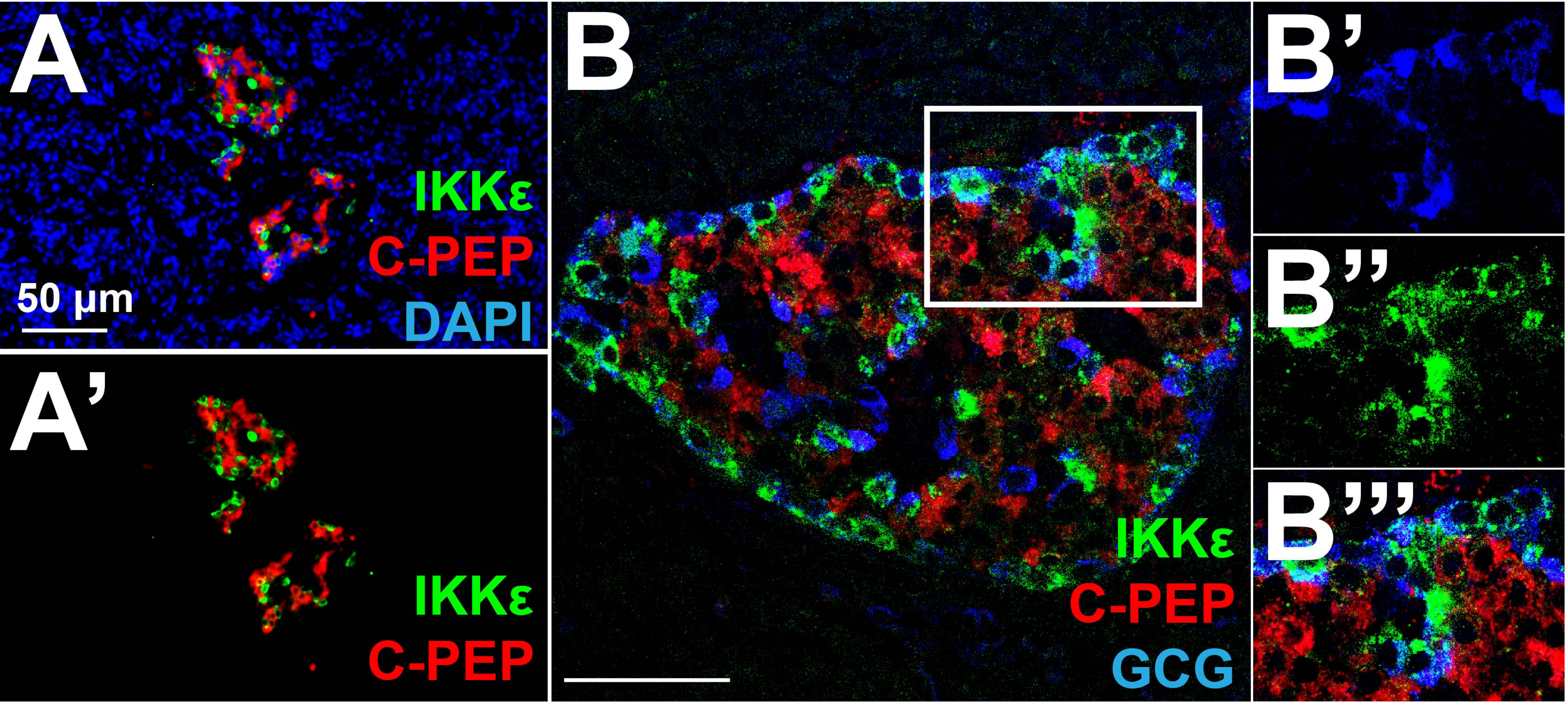

Supplementary Figure 2

C

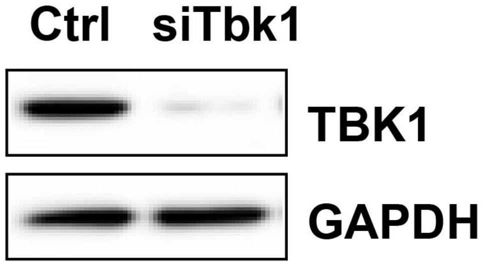

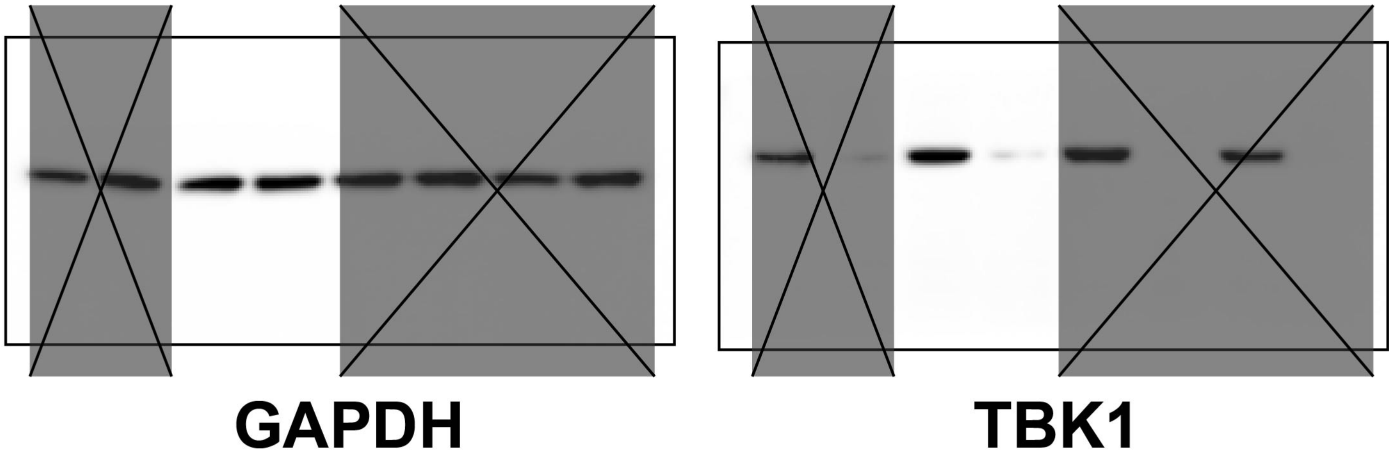

Supplementary Figure 3

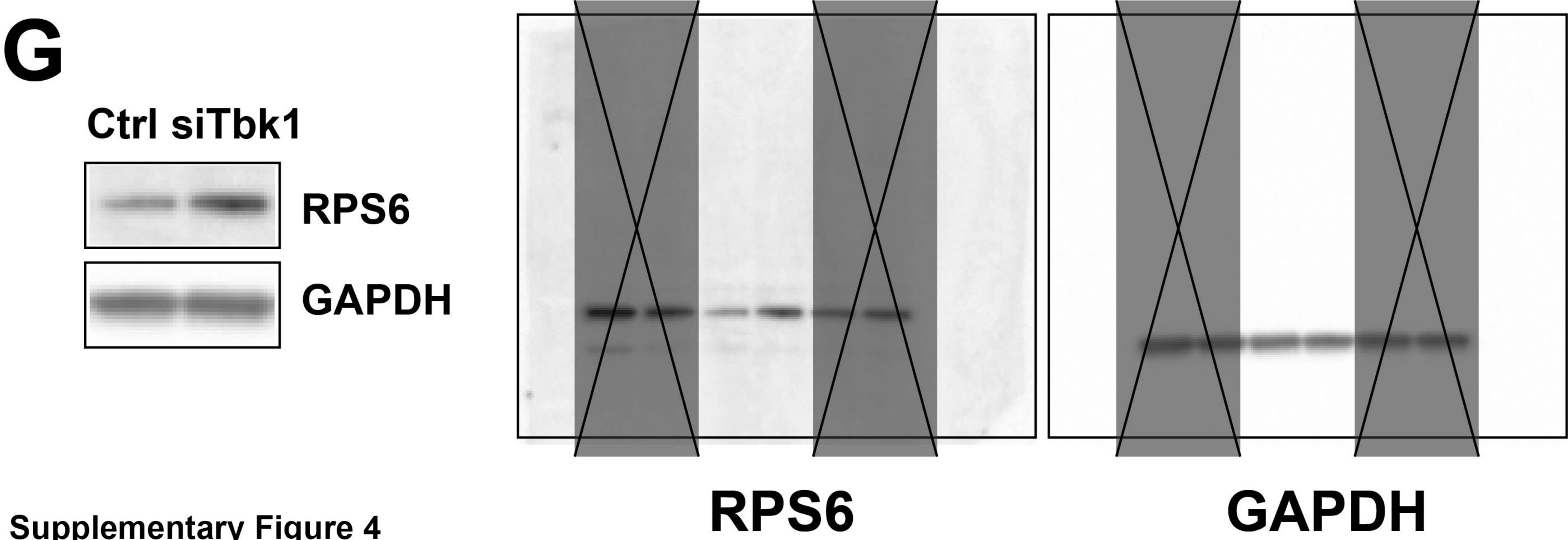

Supplementary Figure 4

A

Proteins		Fold Change	P-value
3-hydroxy-3-methylglutaryl coA synthase 1	HMGCS1	-3.36	3.192E-05
NADH:ubiquinone oxidoredcutase subunit B10	NDUFB10	-1.74	3.184E-04
ATPase H+ transporting V1 subunit E1	ATP6V1E1	-1.68	1.009E-05
ATPase H+ transporting V1 subunit A	ATP6V1A	-1.64	1.885E-05
ATPase H+ transporting V1 subunit B2	ATP6V1B2	-1.53	1.654E-03
Solute carrier family 25 member 10	SLC25A10	-1.50	1.673E-04

B

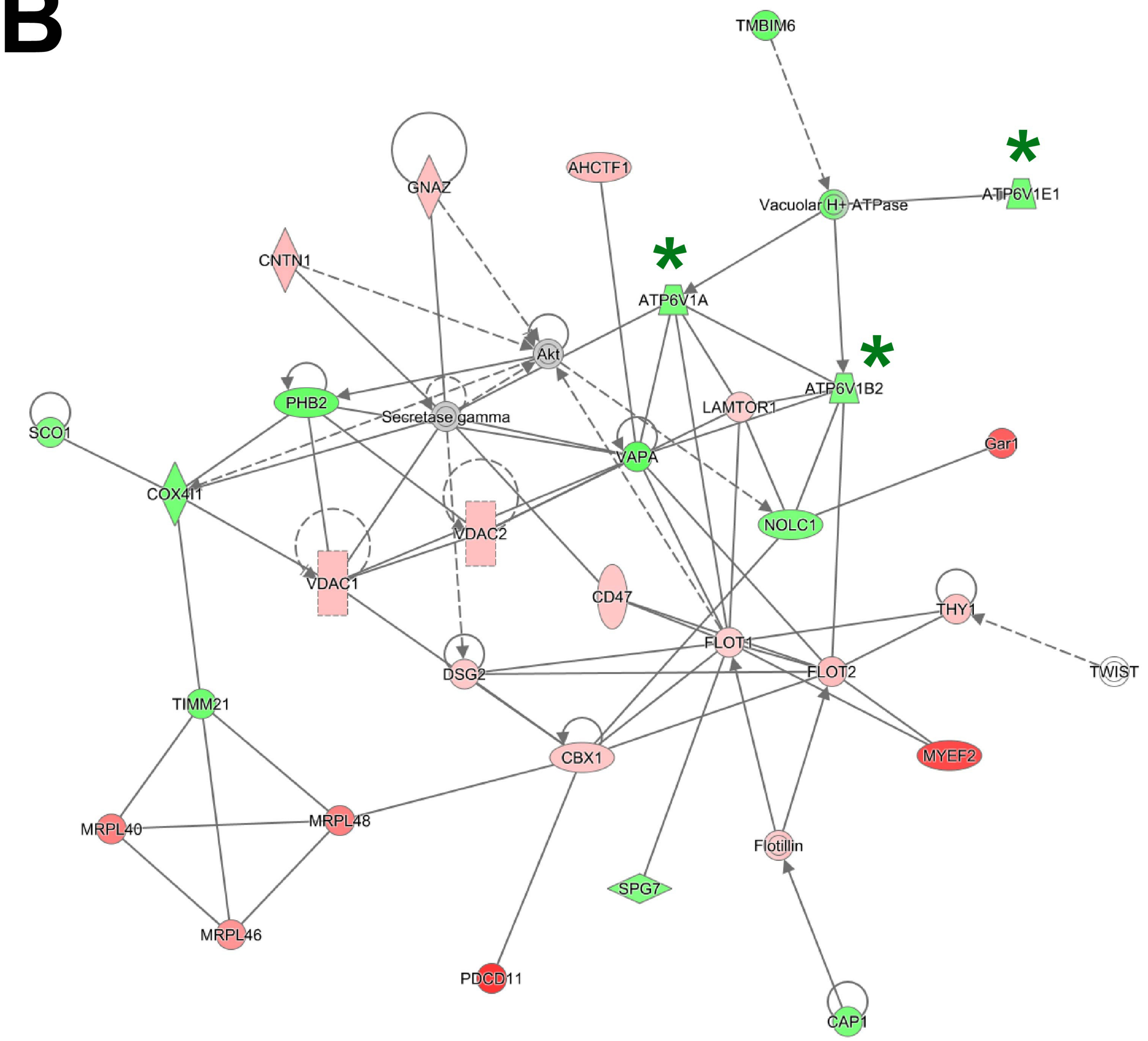

C

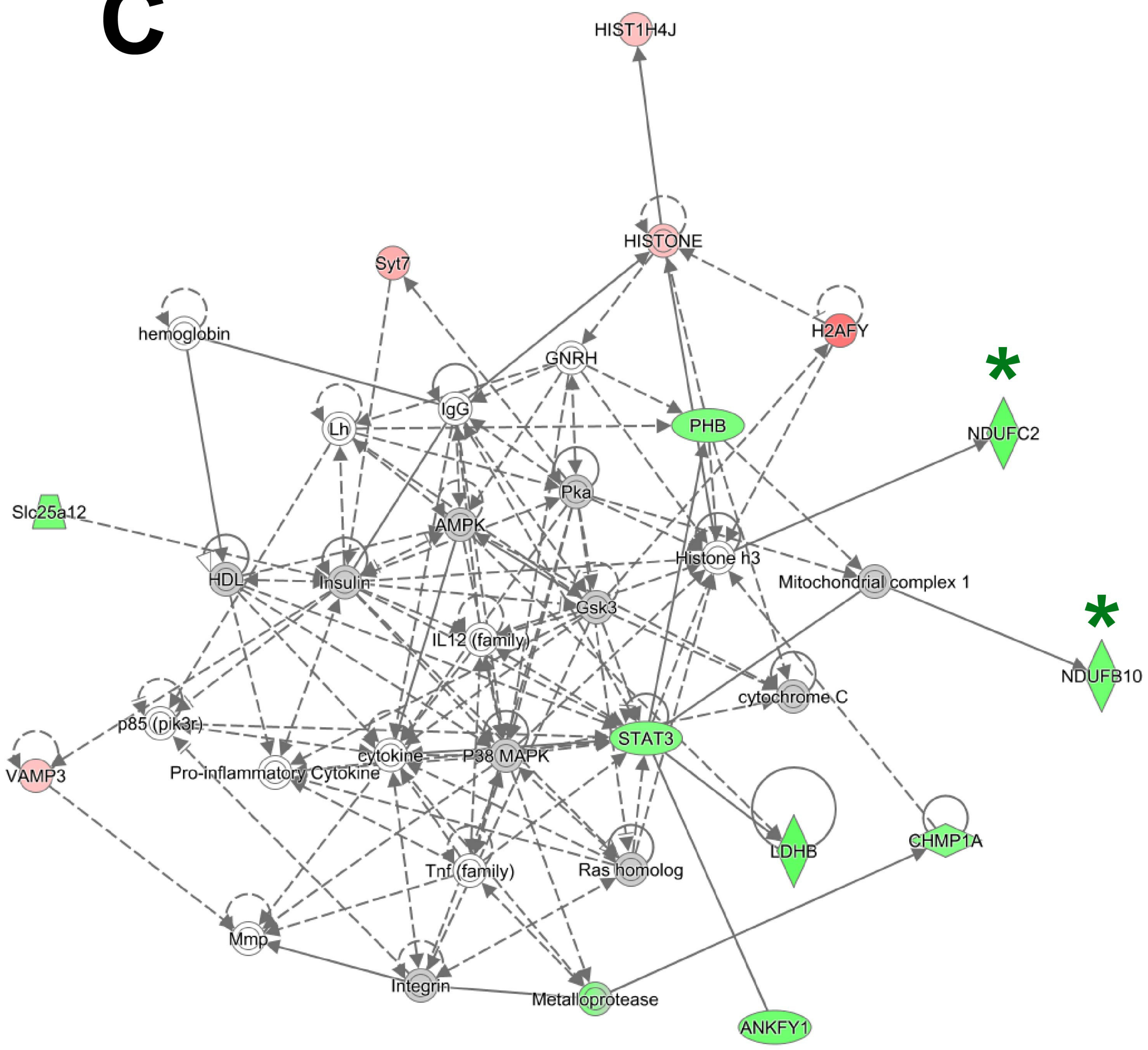

Supplementary Figure 5

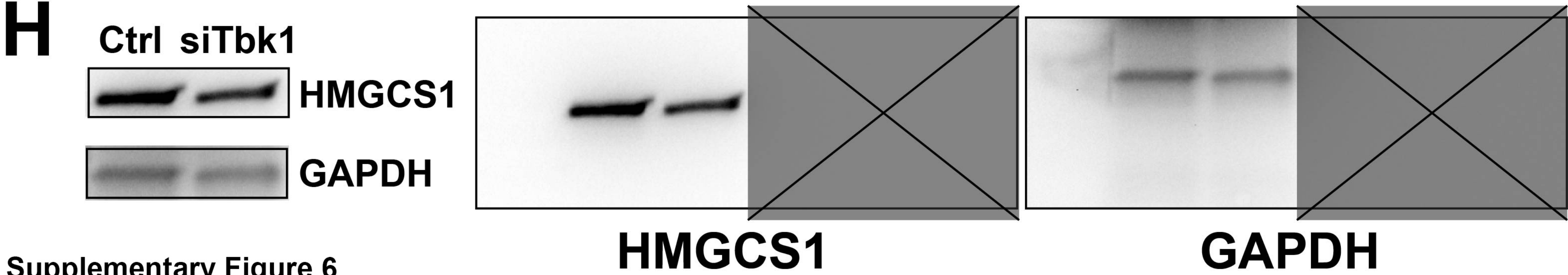

Supplementary Figure 6

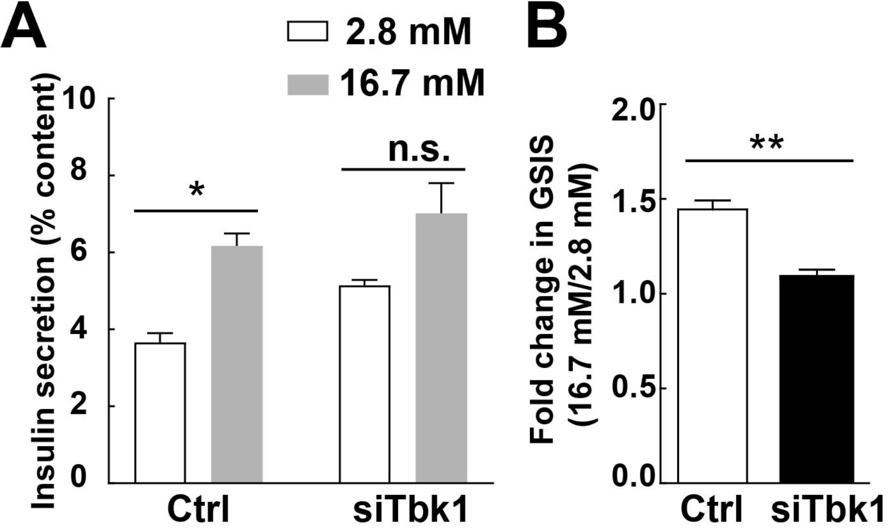

Supplementary Figure 7

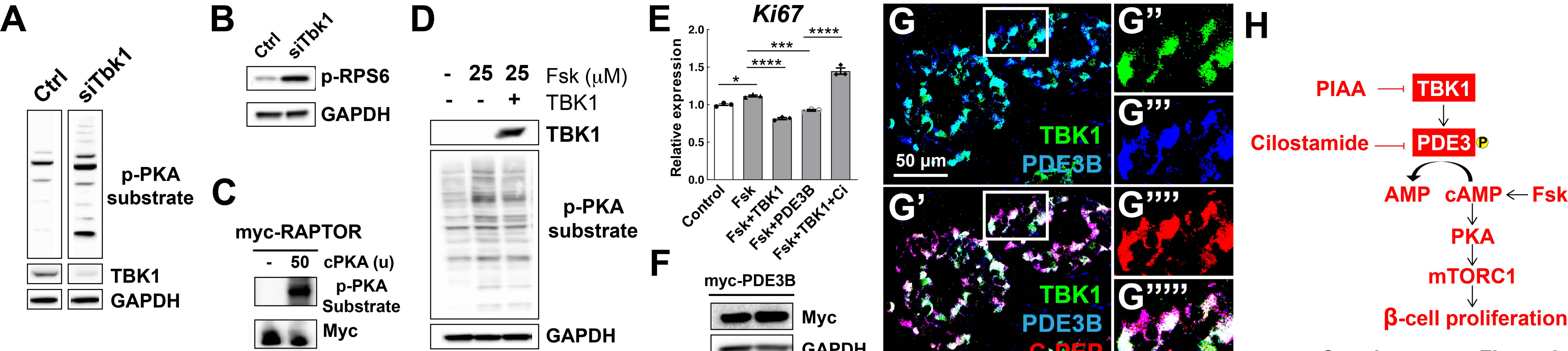

Supplementary Figure 8

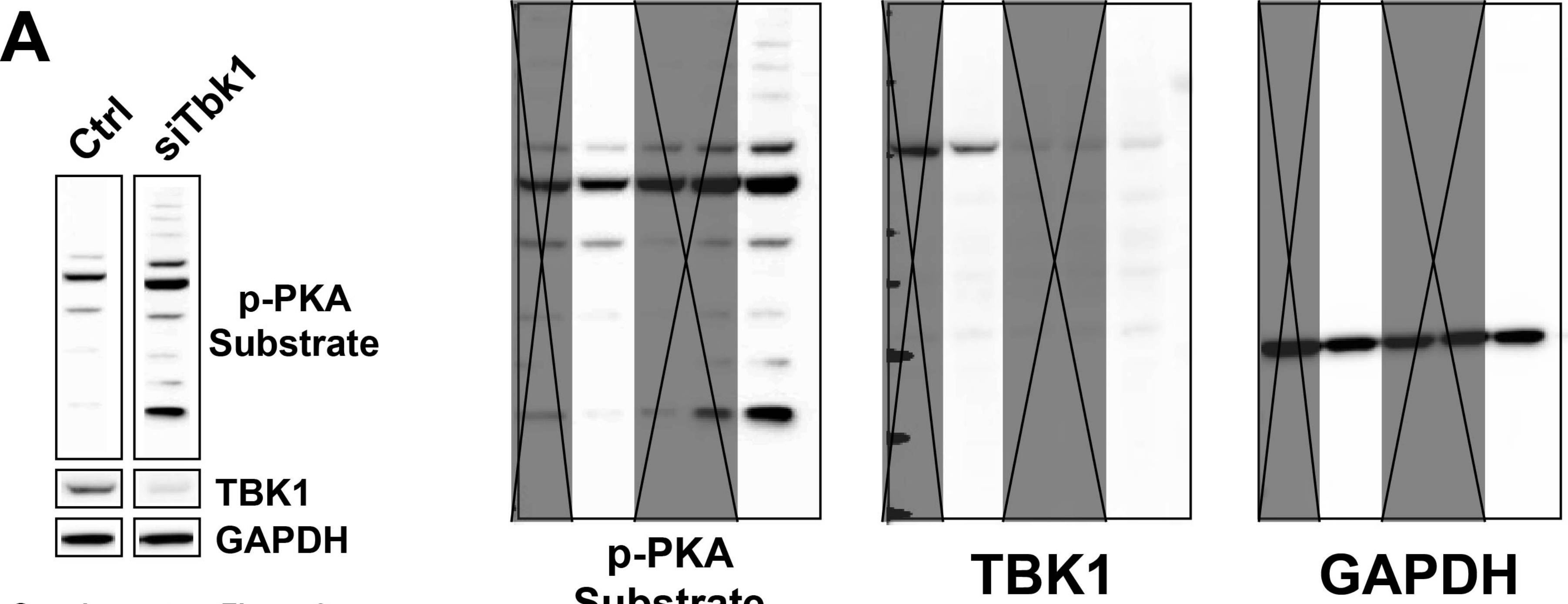

Supplementary Figure 9

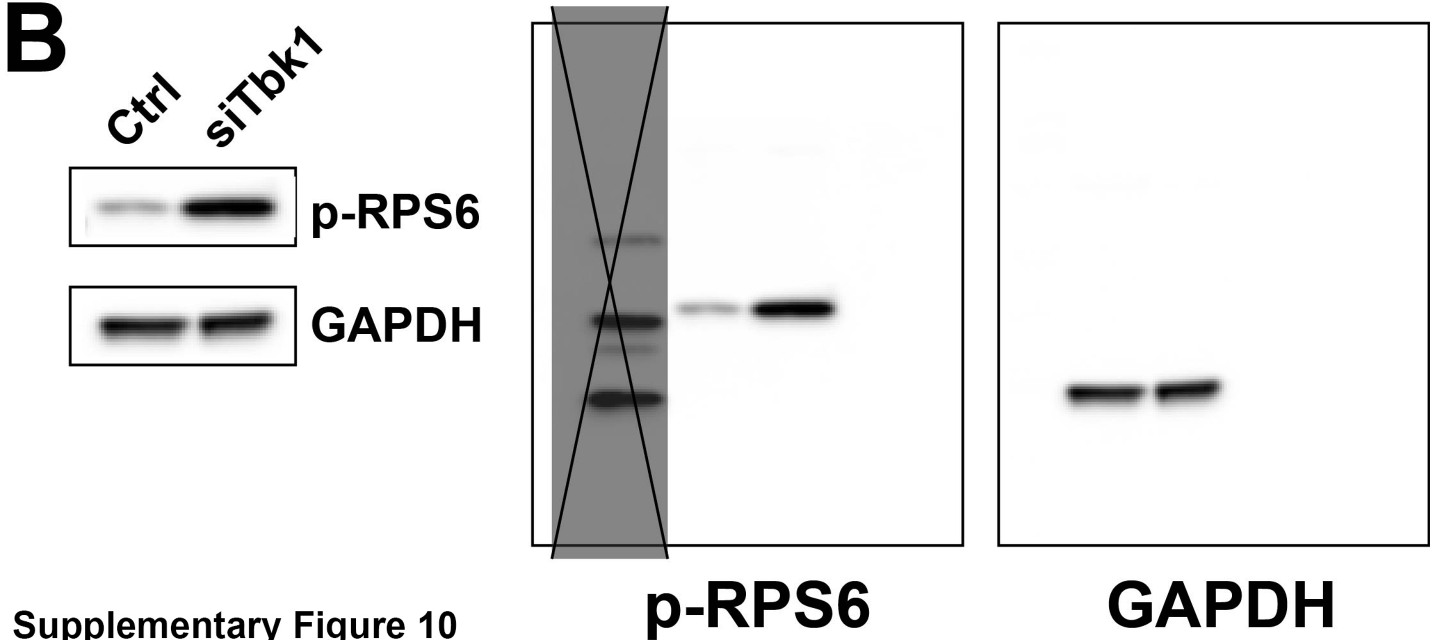

Supplementary Figure 10

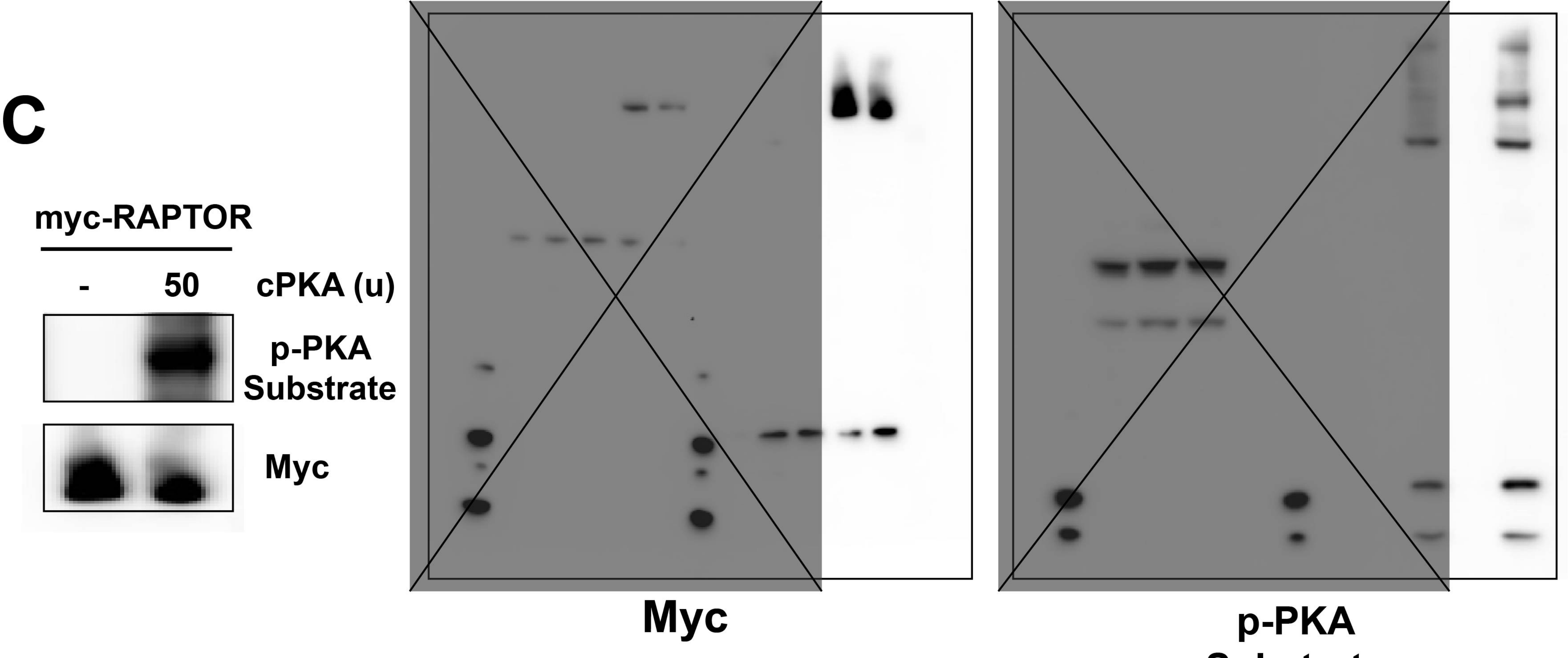

Supplementary Figure 11

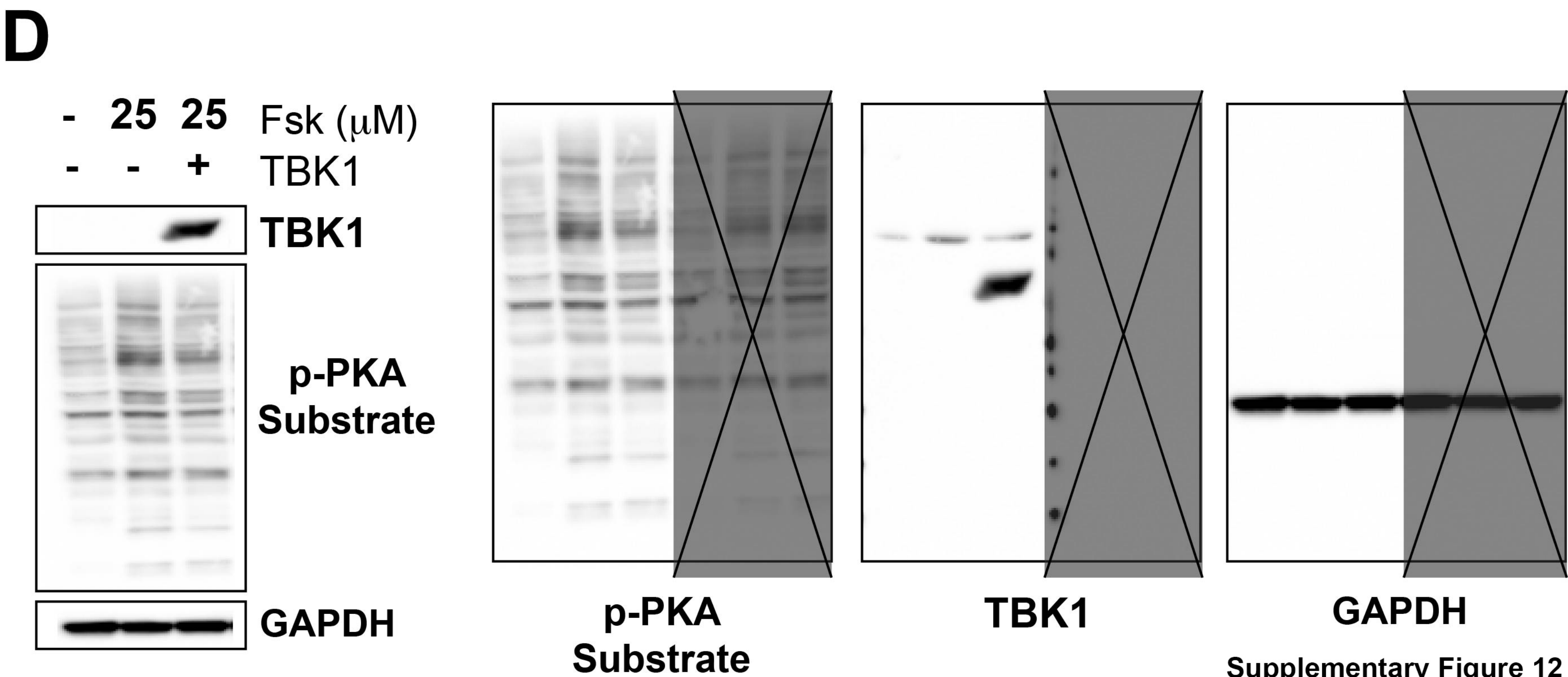

Supplementary Figure 12

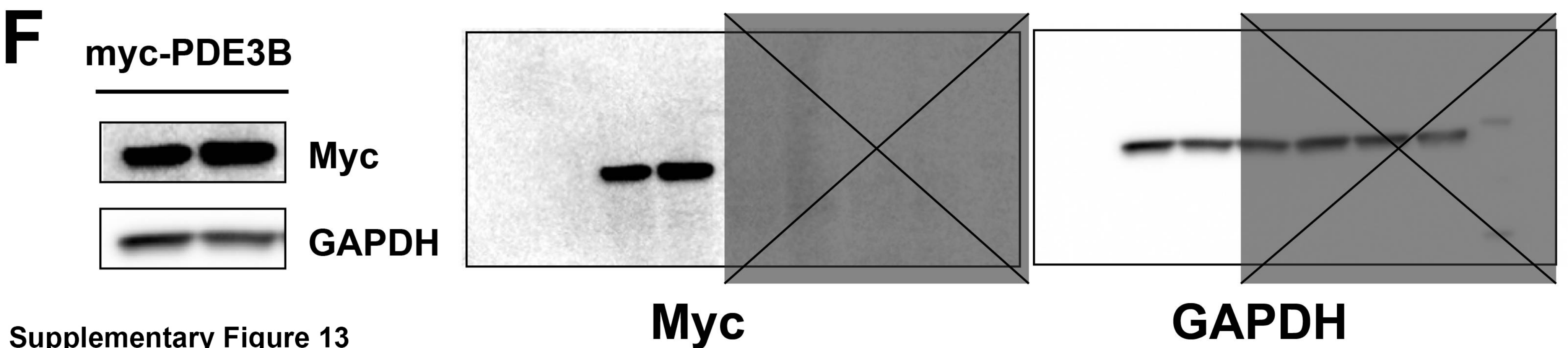

Supplementary Figure 13

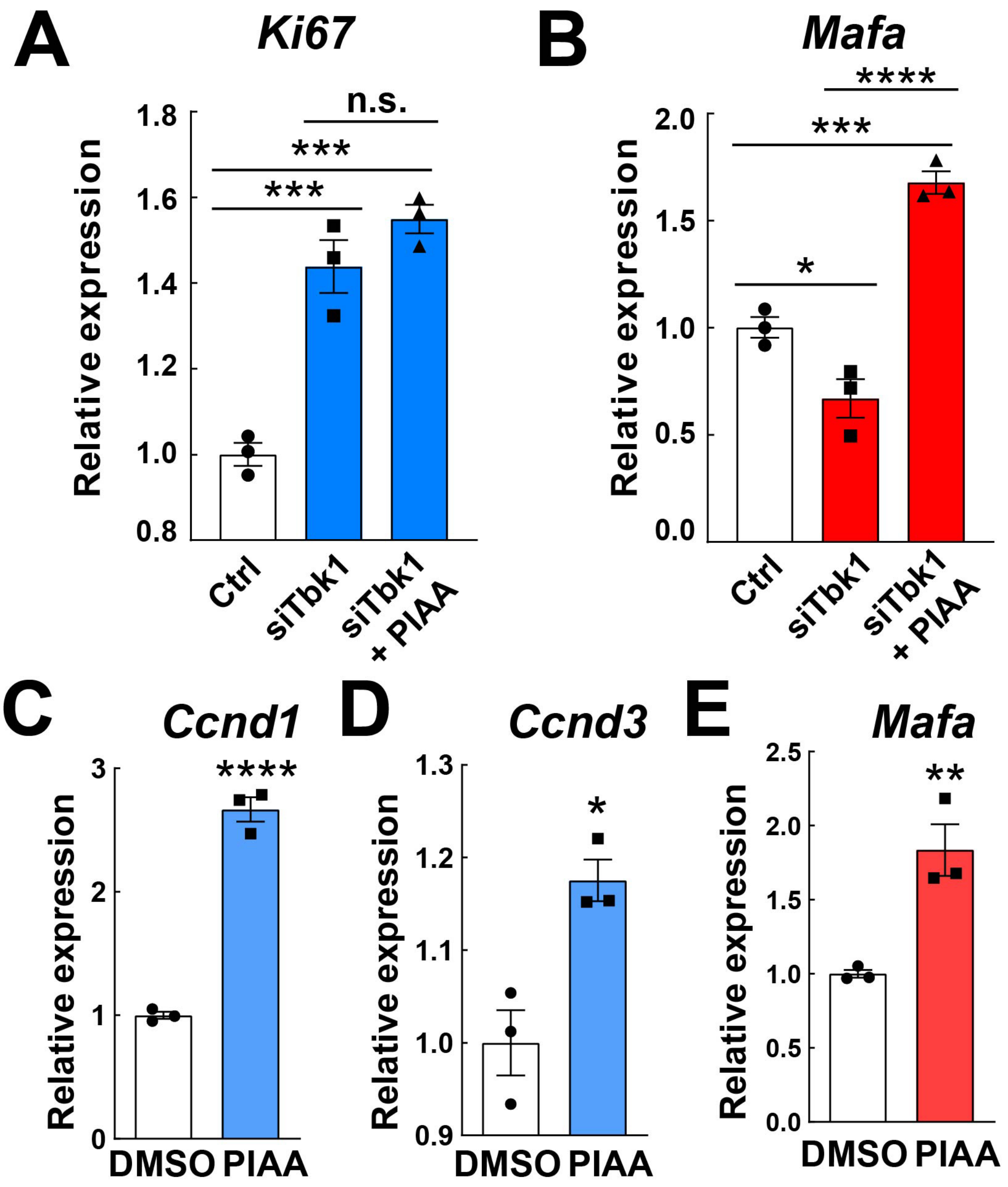

Supplementary Figure 14

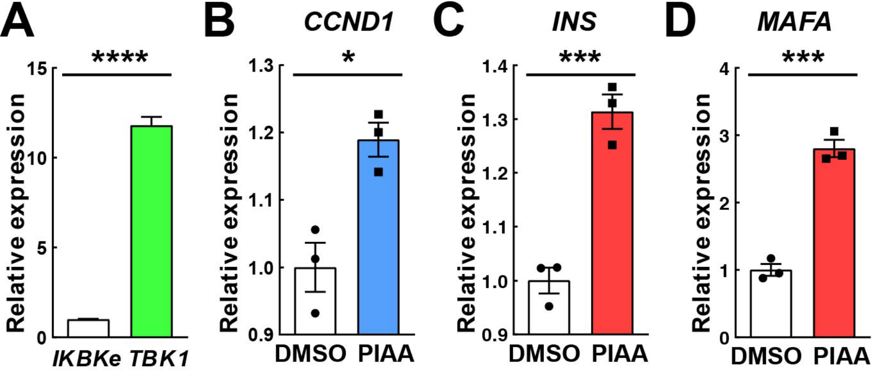

Supplementary Figure 15

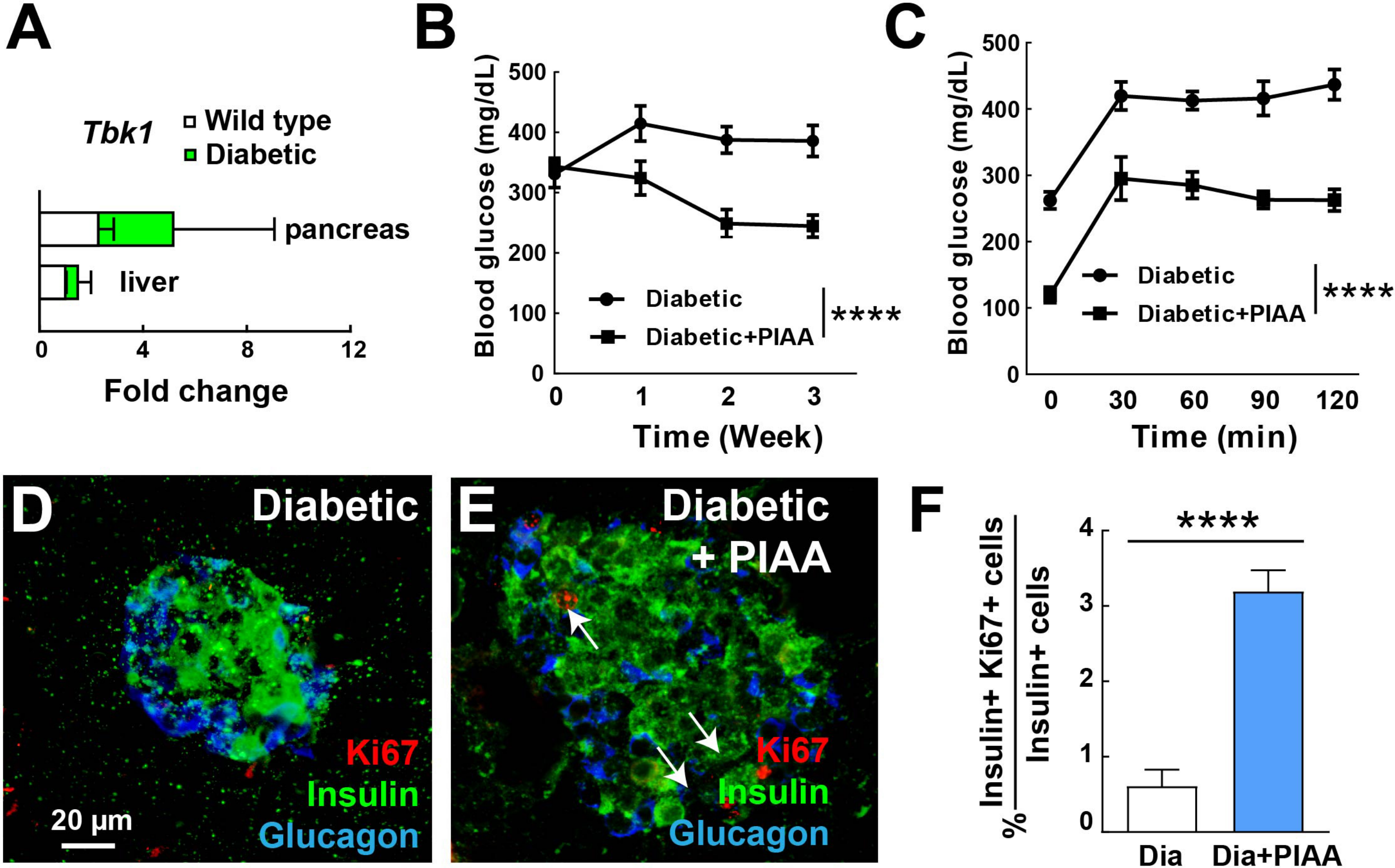

Supplementary Figure 16

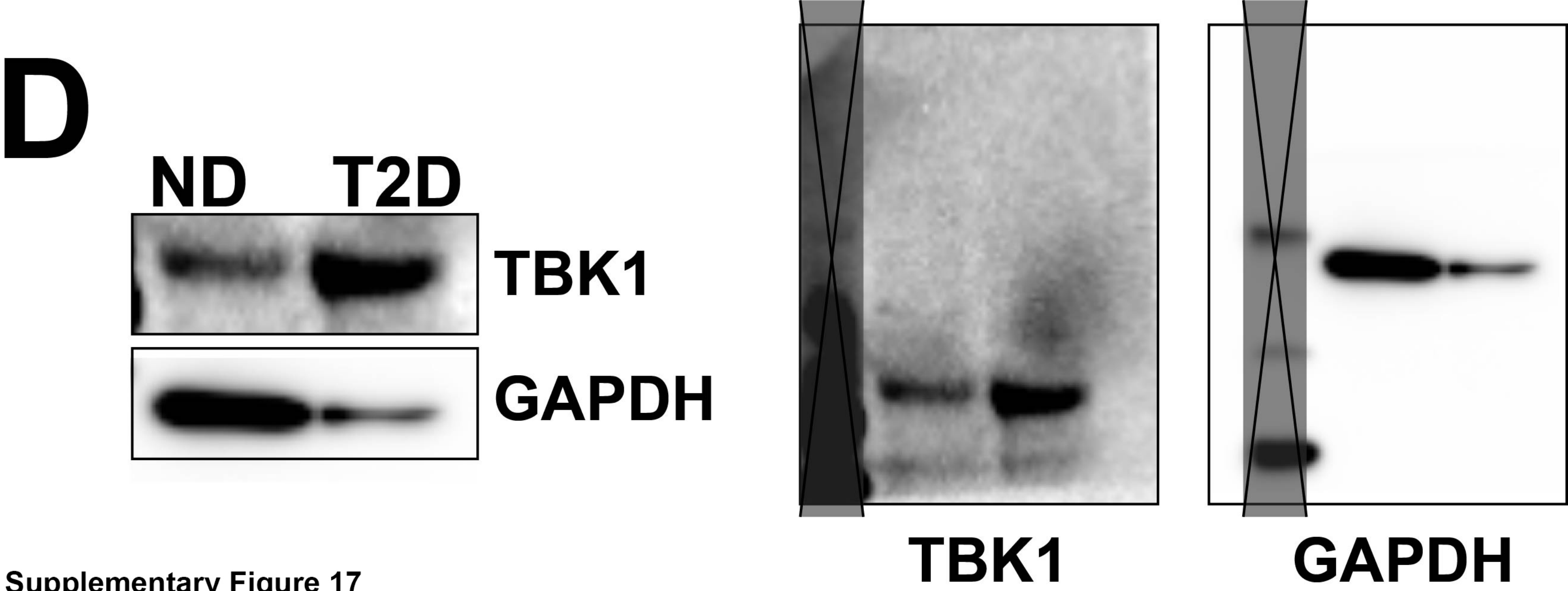

Supplementary Figure 17

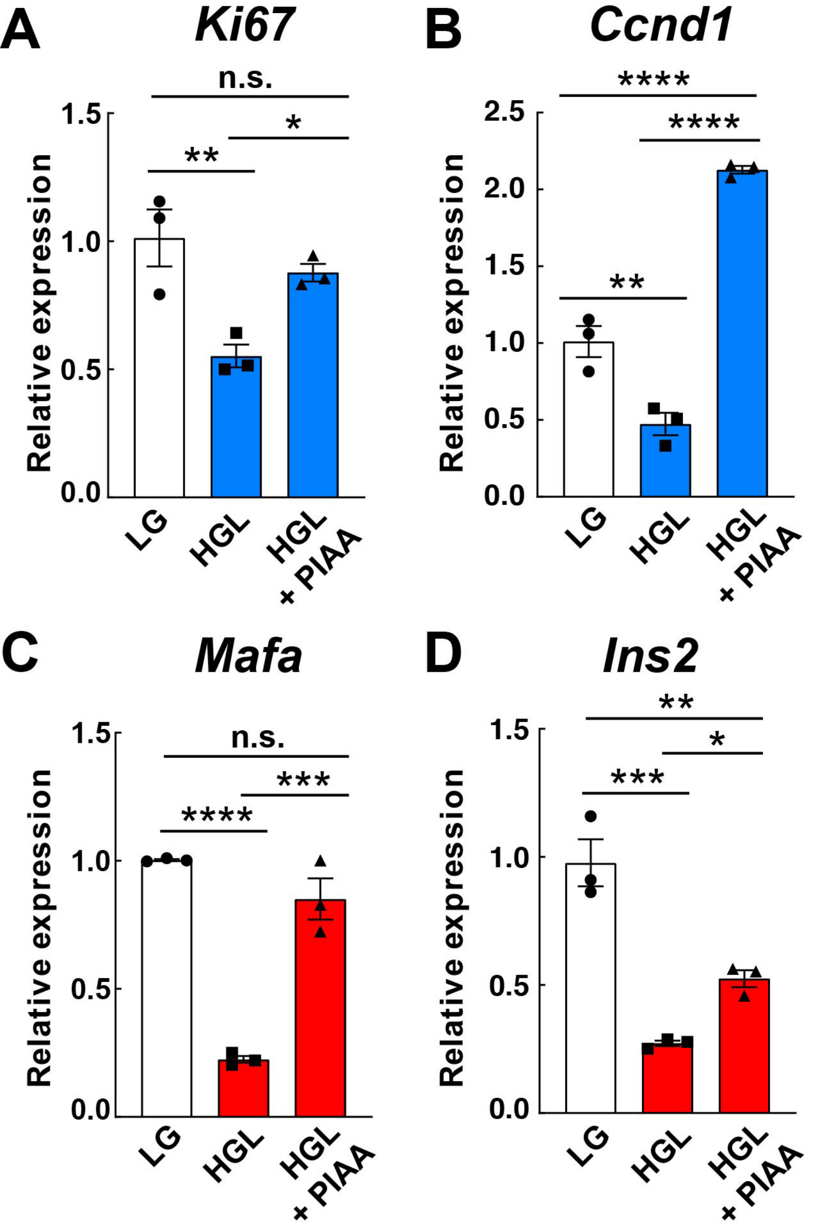

Supplementary Figure 18
